# Comparative Analysis of Proteins Regulated during Cadmium Sulfide Quantum Dots Response in *Arabidopsis thaliana* Wild Type and Tolerant Mutants

**DOI:** 10.3390/nano11030615

**Published:** 2021-03-01

**Authors:** Valentina Gallo, Andrea Zappettini, Marco Villani, Nelson Marmiroli, Marta Marmiroli

**Affiliations:** 1 Department of Chemistry, Life Sciences and Environmental Sustainability, University of Parma, 43123 Parma, Italy; valentina.gallo@unipr.it (V.G.); nelson.marmiroli@unipr.it (N.M.); 2 Institute of Materials for Electronics and Magnetism (IMEM), National Research Council (CNR), 43124 Parma, Italy; andrea.zappettini@imem.cnr.it (A.Z.); marco.villani@imem.cnr.it (M.V.); 3 The Italian National Interuniversity Consortium for Environmental Sciences (CINSA), 43123 Parma, Italy

**Keywords:** proteomics, engineered nanomaterials, mutants, 2D-PAGE, stress response proteins, network analysis

## Abstract

In previous work, two independent *Arabidopsis thaliana* Ac/Ds transposon insertional mutant lines, *atnp01* and *atnp02,* were identified that showed a higher level of tolerance than the wild type (wt) line to cadmium sulfide quantum dots (CdS QDs). The tolerance response was characterized at physiological, genetic and transcriptomic levels. In this work, a comparative analysis was performed on protein extracts from plantlets of the two mutants and of wt, each treated with 80 mg L^−1^ CdS QDs. A comparative protein analysis was performed by 2D-PAGE, and proteins were characterized by MALDI-TOF/TOF mass spectrometry. Of 250 proteins identified from all three lines, 98 showed significant changes in relative abundance between control and CdS QD-treated plantlets. The wt, *atnp01*, and *atnp02* control-treated pairs respectively showed 61, 31, and 31 proteins with differential expression. The two mutants had a different response to treatment in terms of type and quantity of up- and downregulated proteins. This difference became more striking when compared to wt. A network analysis of the proteins differentially expressed in *atnp01* and *atnp02* included several of those encoded by putative genes accommodating the transposons, which were responsible for regulation of some proteins identified in this study. These included nifu-like protein 3 (Nfu3), involved in chloroplast assembly, elongator complex 3 (Elo3), involved in transcriptional elongation, magnesium-chelate subunit-2 (Chli2), involved in chlorophyll biosynthesis, and protein phosphatase 2C (PP2C) which mediates abiotic stress response.

## 1. Introduction

One result of the expansion of nanotechnology has been the large-scale production of engineered nanomaterials (ENMs), which are becoming widely diffused as industrial products for everyday use. However, ENMs can be released into the environment through the recycling of waste, manufacturing, and deliberate or accidental environmental release [1]. Soil is becoming more frequently contaminated with released ENMs, some types of which, due to their small size and high surface reactivity, may enter into plant cells, where both detrimental and positive effects have been noted [2]. Among the smallest ENMs are colloidal cadmium sulfide quantum dots (CdS QDs). The market for colloidal quantum dots (QDs) alone is projected to increase from the current $3 billion to $8.5 billion by 2023, an almost three-fold increase in production and marketing (https://www.marketsandmarkets.com/Market (accessed on 2 February 2021)). QDs are semiconductor nanocrystals measuring around 2–10 nm [3], and their chemical and physical properties are closely size-dependent. Based on their excellent optical properties, QDs have important applications, with development of platforms for simultaneous imaging, sensing, and therapy [4,5]. Indeed, QDs are pivotal constituents of innovative nanotechnology-enabled tools for medical diagnostic and ex vivo imaging [6,7]. QDs have also been used to improve resolution in TVs, digital cameras, computers, and smartphone displays and to increase energy conversion efficiency in quantum dot light-emitting diodes (QLEDs) and in quantum solar cells (QDSSCs) [8,9]. Consequently, the increasing use of QDs-enabled products is expected to result in larger releases of these materials into the environment [10,11]. CdS QDs are very stable and have high reactivity and high surface charge [12]. However, different properties have been connected with CdS QDs toxicity; some studies have implicated Cd release as the main toxicity factor, but under other circumstances, particle properties like shape and size seem to be more relevant [13,14]. Our previous work showed that only about 2% of CdS QDs dissolved in MS medium in 21 days [14]. The main toxicity mechanisms of CdS QDs nanoparticles within a cell or an organism are the generation of reactive oxygen species (ROS), which damage cell membranes, proteins and DNA; the production of protein coronas, removing proteins important to normal cell metabolism [15]; inhibition of the functionality of chloroplasts and mitochondria [16,17]; and the induction of apoptosis through the activation of post-translational regulatory mechanisms such as miRNA [18].

Generally, the influence of exposure duration on Cd-based QDs toxicity is insufficiently understood [19,20]. Only a limited number of studies have analyzed and compared the impacts of CdS QDs and Cd ions on whole organism development, as well as on organ, tissue or cellular structure [21,22,23,24]. Marmiroli et al. [14,25] characterized the transcriptomic and changes associated with exposure to CdS QDs in *A. thaliana*; two mutants (*atnp01* and *atnp02*) of Landsberg *erecta*, collection Soll-Jonson, with Ac/Ds maize transposon insertion, were identified as tolerant to CdS QDs but not to Cd^2+^. Genotypic characterization of *atnp01* and *atnp02* was performed; Southern hybridization profiles based on either EcoRI or BamHI digestion identified two hybridizing fragments in *atnp01,* and a single different one in *atnp02*, demonstrating that the two mutants harbored three different Ds elements. The number of Ds elements inserted in each mutant and their genomic locations were determined by genome walking. Upon sequencing the amplicons produced in the genome walking, a BLAST-based alignment with the full genome sequence identified that the candidate genes potentially affected by the Ds transposition events in *atnp01* were *At3g46880*, coding an unknown chloroplast-localized protein, *At1g13870* (*DRL1*, deformed roots and leaves) coding a cytoplasm-localized calmodulin-binding protein possibly involved in leaf development and meristem structural organization, and *At1g13880* (*ELM2*), a member of the MYB transcription factors category. The *atnp02* event could have affected either *At3g24330*, which encodes an O-glycosyl hydrolase localizing to the endomembrane, or *At3g24430* (*HCF101*), coding for a chloroplast-localizing ATP-binding protein. The Ds element lay within the *At3g24400* pseudogene (*AtPERK2*), which may code for a proline-rich extensin-like receptor kinase.

These mutants were characterized at physiological, genetic, and transcriptomic levels, and a comparison with the response to Cd^2+^ demonstrated the nano-specific nature of the molecular mechanisms and the pathways involved in the tolerance. It was proved by Marmiroli et al. [26] that CdS QDs once inside the plant cell undergo biotransformation rendering them less active and diminishing their power to hamper photosynthesis, respiration, and DNA transcription; this occurs through the inactivation of specific enzymes and by the chaperone action of hard corona proteins, including some heat shock proteins (Hsp70) [15]. The correspondence between transcriptomics and proteomics results for the same toxicant is typically in the range 20–80% [25,27], but proteomics furnishes important information about the cellular response to toxicants, protein–protein interactions, and post-translational modifications that cannot be detected by transcriptomics. Proteins differentially expressed in the presence and absence of CdS QDs were identified by 2D-PAGE. Image analysis was used to compare spots between gels of treated and untreated wt and mutants (*atnp01*, *atnp02*), and those with different intensities were excised and fingerprinted using MALDI-TOF/TOF mass spectrometry. Results obtained with 2D-PAGE were then compared with those obtained in previous work [25], which used the same conditions of treatment but a different separation technique: two-dimensional protein fractionation (ProteomeLab PF2D), employing a gradient isoelectric focusing (IEF) separation in the first dimension, and high-performance liquid chromatography (HPLC) in the second dimension. These two techniques use a different pH range (5–8 vs. 4–8.5), a different solubilization for proteins (SDS vs. urea), and a different quantity of proteins (4 mg vs. 400 µg); therefore, they are often seen as complementary [28]. However, other proteomic techniques, like iTRAQ, have been shown to give a greater number of identifications [29].

## 2. Materials and Methods

### 2.1. Plant Material

*A. thaliana* accession Landsberg *erecta* (L. Heyn) mutants *atnp01* and *atnp02* were previously isolated by screening for resistance to CdS QDs 378 transposon insertional lines obtained from the Nottingham Arabidopsis Stock Centre (uNASC; http://arabidopsis.info/ (accessed on 2 February 2021)). Marmiroli et al., [14] reported the conditions of isolation and characterization of the two mutants when treated with CdS QDs and CdSO_4_. All reagents and standards were purchased from Sigma-Aldrich (St. Louis, MO, USA) unless otherwise stated.

### 2.2. Synthesis and Characterization of the CdS QDs

The synthesis and characterization of CdS QDs are reported in “Supplementary Materials S.1” (Figures S1 and S2). X-ray diffraction (XRD) (XRD Empirean alpha1, Malvern Panalytical, UK) and high-resolution transmission electron microscopy (HR-TEM) (Hitachi HT7700, Hitachi High Technologies America, Pleasanton, CA, US) demonstrated that the average static diameter of the CdS QD NPs (nanoparticles) was 5 nm; the crystal structure was hexagonal wurtzite (ZnS) with about 78% Cd. The average particle size of the aggregates measured by dynamic light scattering, and zeta potential (ζ) estimated in ddH_2_O, were 178.7 nm and + 15.0 mV, respectively [14].

### 2.3. Seed Germination, Growth, and Treatments

Twenty-five seeds of *A. thaliana* wild type (wt) and two mutants, *atnp01* and *atnp02*, were sown on Petri dishes containing Murashige and Skoog (MS) nutrient medium (Duchefa Biochemie, Haarlem, Netherlands) containing 1% *w*/*v* sucrose and 0.8% *w*/*v* agar, then placed in the dark, under controlled conditions in a growth chamber. After germination, seedlings were grown at 24 °C, with a relative humidity of 30%, and under a 16-h photoperiod (light intensity 120 μM m^−2^ s^−1^ photosynthetic photon flux) in the MS medium in the absence of treatment for 14 days. After that time span, seedlings were transferred to a fresh MS medium containing 80 mg L^−1^ CdS QDs (treatment) or without CdS QDs (control) and grown for a further 21 days in the same conditions as above. Because 80 mg L^−1^ CdS QDs is a sub-inhibitory concentration, the stocks of seeds of both mutants and wt plants were checked for their capacity to grow in the presence of CdS QDs up to 250 mg L^−1^, which was totally inhibiting for wt. All six samples were collected after 21 days by removing them carefully from the medium; they were gently washed with distilled H_2_O, frozen in liquid nitrogen and then stored at −80 °C until use for protein extraction.

### 2.4. Protein Extraction and Quantification

Proteins of wt and of the two mutant lines, untreated and treated (80 mg L^−1^ CdS QDs), were extracted. Frozen samples were ground to powder in liquid nitrogen using a mortar and pestle; 1 g aliquot was suspended in 6 mL of extraction buffer: 700 mM sucrose, 500 mM Tris-HCl, pH 7.5, 50 mM ethylenediaminetetraacetic acid (EDTA), 100 mM KCl, 2% dithiothreitol (DTT), 0.1% Protease Inhibitor Cocktail (Sigma-Aldrich), vortexed and mixed for 10 min on ice. An equal volume of 500 mM Tris-HCl buffered phenol was added, and the solution was mixed at room temperature for 10 min [30,31]. The samples were centrifuged for 10 min at 5500× *g* and at 4 °C. The phenolic phase was collected in a new tube and back-extracted with 3 mL of extraction buffer. Proteins were precipitated from the phenolic phase overnight at −20 °C by adding five volumes of 0.1 M ammonium acetate (J.T. Baker, Deventer, The Netherlands) saturated in methanol. Precipitated proteins were centrifuged for 30 min at 5500× *g* and at 4 °C, the pellet was washed with cooled methanol and then with cooled acetone. After each washing step, the sample was centrifuged for 5 min at 5500× *g* and at 4 °C. Finally, the pellet was dried using a Speed Vac Concentrator 5301 (Eppendorf AG, Barkhausenweg, Hamburg, Germany).

The pellet was dissolved in 300 µL of isoelectofocusing (IEF) buffer containing 9 M urea, 4% 3-[(3-cholamidopropyl) dimethylamino]-1-propanesulfonate (CHAPS), 50 mM DTT, 0.001% protease inhibitor cocktail, 1% carrier ampholyte mixtures (pH 3–10, BioRad, CA, USA). Protein quantification was evaluated according to a modified Bradford assay [32] based on the acidification of the sample buffer with 20 mM HCl. Bovine serum albumin (BSA) was used as standard.

### 2.5. 2D Gel Electrophoresis

The proteins mixtures were resolved by two-dimensional polyacrylamide gel electrophoresis (2D-PAGE). For proteins separation in the first dimension (IEF), 400 µg of proteins of each sample were loaded onto 11-cm ReadyStrip pH 5-8 IPG strips (BioRad, CA, USA) which had been rehydrated overnight with 250 µL IEF buffer containing the sample. Proteins were focused by PROTEAN^®^ i12™ IEF System (BioRad, CA, USA) applying 250 V (60 min), 1000 V (60 min), 8000 V (2 h) and 8000 V up to 35,000 V/h.

After IEF, the strips were incubated 15 min in 3 mL of reducing buffer containing 6 M urea, 2% *w*/*v* DTT, 0.375 M Tris-HCl (pH 8.8), 20% *w*/*v* glycerol, 2% *w*/*v* SDS and for 15 min in 3 mL of alkylating buffer containing 6 M urea, 2.5% w/v iodoacetamide, 0.375 M Tris-HCl (pH 8.8), 2% *w*/*v* glycerol. The second dimension (SDS-PAGE) was performed using a CriterionTM Dodeca™ cell (BioRad, CA, USA) and 12% Criterion™ XT Bis-Tris gels (BioRad, USA) in 1 M MOPS (3-(N-morpholino)-propanesulfonic acid) buffer (1 M Tris, 20 mM EDTA and 2% *w*/*v* SDS).

2D gels were stained with QC Colloidal Coomassie G-250 (BioRad, CA, USA) and were scanned with a ChemiDocTM Imaging System (BioRad, CA, USA). Gel analysis was performed using PDQuest software (BioRad, CA, USA). Spot detection and matching between gels were performed automatically, followed by manual verification. The spot densities were normalized by local regression method and followed by a calculation against the whole gel densities. The percentage density of every spot was averaged over three replicate gels, and Student’s *t*-test analysis (*p* < 0.05) was carried out to find statistically significant differences in protein abundances. Statistically significant spots were then excised from the gels using an EXQuest Spot Cutter (BioRad, CA, USA), destained by soaking the pieces of acrylamide in a 1:1 solution of 100 mM ammonium bicarbonate/acetonitrile for 30 min, and the proteins were hydrolyzed with trypsin at 37 °C overnight [33].

### 2.6. MALDI-TOF/TOF Mass Spectrometry

The solutions containing the tryptic peptides were desalted and concentrated to a final volume of 4 µL with Zip-Tip C18 (Millipore Corporation, Billerica, MA, USA), according to the manufacturer’s protocol, then dispersed into an α-cyano-4-hydroxycinnamic acid (4-HCCA) matrix, prepared by dissolving 4-HCCA in 50% acetonitrile/0.05% trifluoroacetic acid and spotted on a MALDI plate. The analysis was performed through a model 4800 MALDI-TOF/TOF^TM^ MS analyzer (Applied Biosystems, Foster City, CA, USA). Peptide mass spectra were acquired in reflectron mode (500–4000 m/z range) and analyzed with the help of mMass v5.5 open-source software (http://www.mmass.org/ (accessed on 2 February 2021)). A peak list was created for each feature, and then manually controlled for the presence of signal from the matrix complex, human keratin peptides and trypsin. The main parameters were set as follows: digestion enzyme trypsin with one missed cleavage, mass type monoisotopic, 100 ppm peptide tolerance, methionine oxidation and cysteine carbamidomethylation were set to enzymatic cleavage as fixed and variable modifications respectively.

Peptide mass fingerprinting analysis was performed with the software Mascot (http://www.matrixscience.com (accessed on 2 February 2021)) and proteins were identified with the Swiss-Prot data base of *A. thaliana* (thale cress). The information about gene loci was found in the UniProt and in TAIR database (https://www.arabidopsis.org/ (accessed on 2 February 2021)) for the corresponding *A. thaliana* proteins names and description.

### 2.7. Data Mining and Analysis

Heat maps of significant proteins were generated by R v3.3.1 (www.r-project.org (accessed on 2 February 2021)). The Gene Ontology enrichment analysis was performed through the Panther database (pantherdb.org/). The pathway analysis was performed using the GoMapMan tool based on ITAG Release 2.3 234 (2011-04-26) of the *A. thaliana* genome sequence. MapMan 3.6.0RC1 software (mapman.gabipd.org/web/guest/mapman-download) was used to place proteins within a likely pathway (BIN). Protein–protein interactions (PPIs) of differentially expressed proteins from *A. thaliana* were performed using STRING v11.0 (https://string-db.org/ (accessed on 2 February 2021)).

## 3. Results and Discussion

### 3.1. A. Thaliana Proteome after CdS QDs Treatment

**Figure 1:**
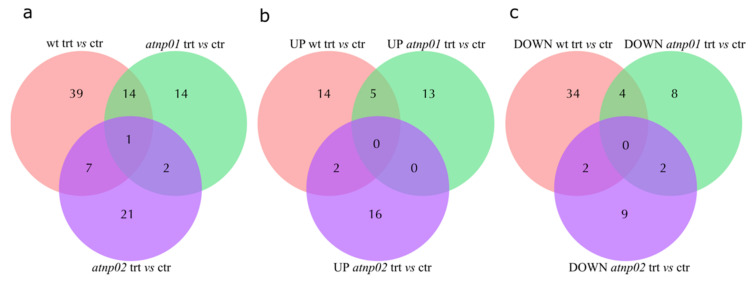
Venn diagrams showing proteins common and non-common to wt and *atnp01*, to wt and *atnp02*, to *atnp01* and *atnp02* and to all treatment conditions. (**a**) There were 15 proteins common between wt and *atnp01*, 8 between wt and *atnp02*, and 3 between the two mutants; (**b**) The proteins upregulated in wt, *atnp01* and *atnp02* lines between control and treatment conditions. There were four upregulated proteins in common between wt and *atnp01*, two between wt and *atnp02*, and none between the two mutants; (**c**) The proteins downregulated in wt, *atnp01* and *atnp02* mutant lines between control and treatment conditions. There were four downregulated proteins in common between wt and *atnp01*, two between wt and *atnp02*, and two between the two mutants.

2D-PAGE profiling (technical triplicates) of the plant proteomes of A. thaliana generated overall about 600 visible protein features for each of the wt and two mutants, exposed or not exposed to CdS QDs. After minor spots were eliminated to allow consistent MALDI-TOF/TOF analysis, about 250 reproducible spots remained (Figure S3). For the control samples, we had a total of 103 reproducible spots, of which 79 were present in all three plants; 2 were found only in wt, and 1 only in *atnp*01, with no unique spots for atnp02 (Figure S4A). For the treated samples, we had 105 reproducible spots, of which 81 were common to all the plant types. A further 4, 0 and 3 were unique to wt, *atnp*01, and *atnp*02 respectively (Figure S4B). The proteins in common between wt and mutants are shown in Figure S4 and explained in the Figure caption. Of these spots, 98 were designated as ‘differentially abundant’, meaning that they varied in intensity with p-value ≤ 0.05 between the treated (trt) and untreated (ctr) plant pairs. Of these, 61 were from the wt pair, 31 from the *atnp*01 pair and 31 from the *atnp*02 pair (Figure 1A). The numbers of commonalities and up- and downregulated proteins between wt and mutants and between mutants are listed in the caption to Figure 1.

**Figure 2:**
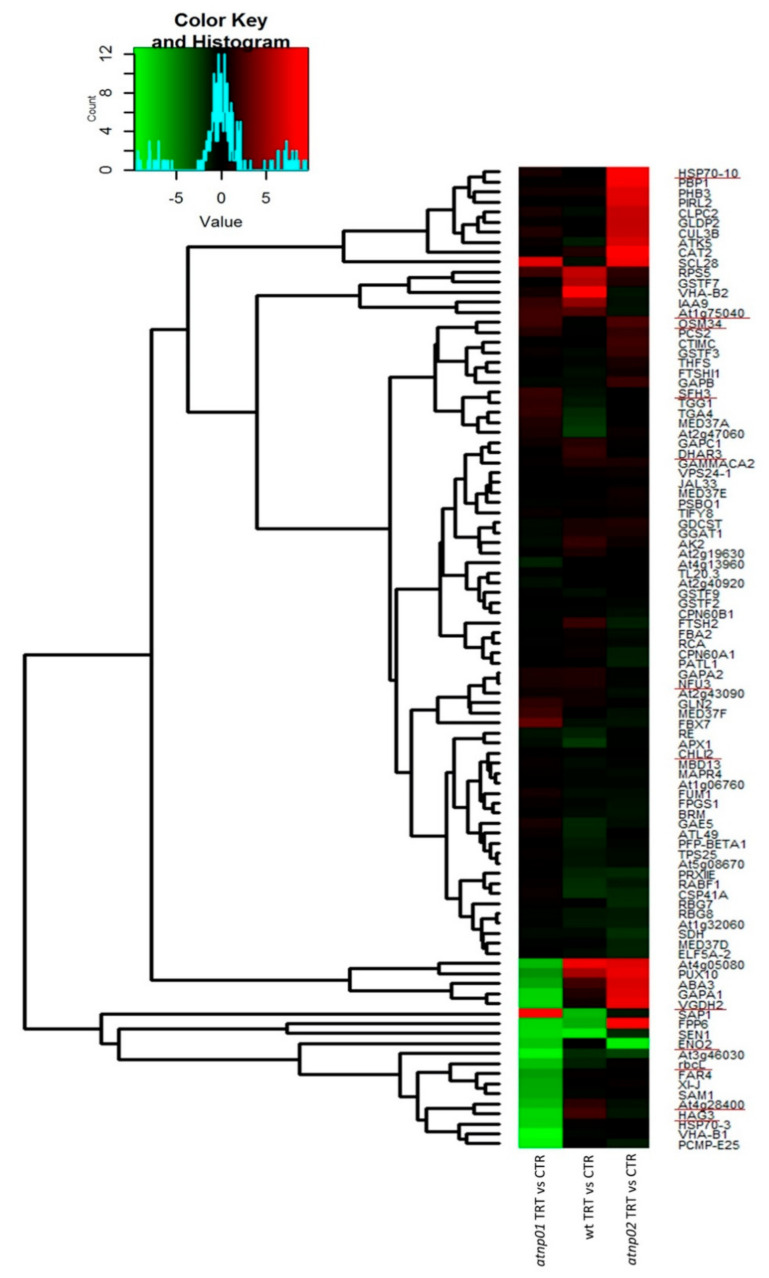
Heat map representing the effect on the A. thaliana proteome of CdS QDs (80 mg L^−1^) for wt and for the mutant lines *atnp*01 and *atnp*02. This heat map was obtained, similarly to Figure S5, by dividing the abundances of treated samples by the control samples. The proteins underlined in red are those in common among the wt and the two mutants *atnp*01 and *atnp*02, those that were found in the networks related to the mutated genes in the two mutants and those in common with the proteomic study with PF2D and the transciptome.

The identities of the differentially abundant proteins are reported in Table S1, and the associated heat map is shown in Figure 2. The heat map was obtained after the computation of the protein abundances in the control and treated samples for all the plant types, and by dividing the treated by the control to obtain the final differential heat map. Figure S4 shows the heat maps for all the treatments and controls of wt, *atnp*01, and *atnp*02. For all 98 proteins, the MapMan ontology BIN assignations are listed in Table S2. Most of the proteins in wt were downregulated, annotating in processes such as biotic and abiotic stress responses, protein folding, and protein degradation (Figure 2). Conversely, in both mutants, there is a balance between the numbers of up- and downregulated proteins (Figure 2). Comparing the heat map of treated vs. control, it can be observed that, whereas the treatment for the wt led to a general down-regulation of proteins, for the two mutants antnp01 and atnp02 there was also a significant number of proteins upregulated (Figure 2). The proteome of each mutant differed from that of the wt, both when the plants were grown under control conditions and when they were exposed to CdS QDs. Only one of the variable features was in common among all comparisons, namely putative protein phosphatase 2C 58 (At4g28400), downregulated in both mutants and upregulated in wt (Figure 1A and Figure 2). This is a protein phosphatase known to mediate abiotic stress pathways and it is a member of major phosphatase class PP2C [34,35]. The phytohormone abscisic acid (ABA) is a major player in the regulation of responses to abiotic stresses, in particular drought and salinity. In Arabidopsis, ABA signaling, triggered particularly under abiotic stresses, involve various PP2Cs members as key regulators [36]. Two proteins were common to both mutants in the treatment condition, namely bifunctional enolase 2/transcriptional activator (Eno2) and putative pectinesterase/pectinesterase inhibitor VGDH2 (Vgdh2) (Figure 1A). Eno2, downregulated in the mutants (Figure 2), is the key glycolytic enzyme and encoded by LOS2 (Low expression of osmotically responsive genes 2). The ENO2 locus is highly expressed throughout plant development and is on average 10-fold more abundant than ENO1 and ENO3 in all tissues and organs [37]. Interestingly, Eno2 is a key protein in glycolysis and might be involved in the response to CdS QDs which can include impaired glycolysis. Vgdh2, downregulated in *atnp*01 and upregulated in *atnp*02 (Figure 2), acts in the modification of cell walls via demethylesterification of cell wall pectin [38].

Separately considering the up- and downregulated proteins, we constructed two new Venn diagrams (Figure 1B,C). The diagram for the upregulated proteins indicates that there are five proteins in common between treated wt and treated atnp01: pathogenesis-related protein 5 (At1g75040), F-box only protein 7 (Fbx7), oxygen-evolving enhancer protein 1-1, chloroplastic (Psbo1), probable fatty acyl-CoA reductase 4 (Far4), and gamma carbonic anhydrase 2 (Gammaca2). Pathogenesis-related protein 5 is partially responsible for acquired pathogen resistance [39]. Fbx7 is required for protein synthesis during temperature stress. [40]. Psbo1 stabilizes the manganese cluster which is the primary site of water splitting [41]. Far4 provides the fatty alcohols required for the synthesis of suberin in roots, seed coat, and wound-induced leaf tissue [42]. Gammaca2 mediates complex I assembly in mitochondria and respiration [43]. There are two proteins in common between treated wt and treated *atnp*02: vacuolar protein sorting-associated protein 24 homolog 1 (Vps24-1) and 30S ribosomal protein S5, chloroplastic (At2g33800). Vps24-1 is required for multivesicular body (MVB) formation and sorting of endosomal cargo proteins into MVBs. [44]. At2g33800 binds directly to 16s ribosomal RNA [45]. In the groups considered, there are no proteins in common between the two treated mutants. Among the downregulated proteins we found four proteins in common between treated wt and treated *atnp*01: heat shock 70 kDa protein 3 (Hsp70-3), histone H2B.7 (At3g46030), L-ascorbate peroxidase 1 (Apx1), and filament-like plant protein 6 (Fpp6). Hsp70-3, in collaboration with other chaperone proteins, assists translocation of precursor proteins into organelles, facilitates folding of de novo synthesized proteins, and is responsible for the degradation of damaged proteins undergoing stress conditions such as from Cd [46]; At3g46030 is a core component of the nucleosome [39]; Apx1 is a key component of the reactive oxygen species gene network, moreover, its synthesis can be induced by Cd exposure [46]. The function of Fpp6 is unknown. We found two proteins in common between treated wt and treated *atnp*02: glycine-rich RNA-binding protein 8 (Rbg8) and Ras-related protein RABF1(Rabf1). Rbg8 plays a role in RNA transcription or processing during stress [47]. Rabf1 is an endosomal protein probably involved in endocytosis [48]. There are two proteins in common between the two treated mutant lines: probable protein phosphatase 2C 58 and enolase 2. The first of these proteins was found in common between the wt and two mutants (Figure 1A), but it was upregulated in the wt while being downregulated in the two mutants (Figure 2). The second protein was found in common between the two mutants and was downregulated.

From the lower part of the heat map (Figure 2), it seems that the majority of the downregulated proteins are specific to the mutant *atnp*01, with the exception of Sap1, a zinc-finger protein and Scl28, a serine-arginine-like splicing factor, both upregulated. The most strongly downregulated proteins are: Pcmp-E25, a pentatricopeptide; Hsp70-3, a heat shock protein; Sam1, a S-adenosylmethionine synthetase; Xi-J, myosione 16; Far4, fatty-acyl-CoA reductase, At3g46030, histone H2B.7, VHA-B1, V-type proton ATPase, SEN1, t-RNA splicing endonuclease, and FPP6, filament like plant protein. Instead, ENO2 and PP2C were downregulated in both mutants. For the proteins Vgdh, Gapa1, Aba3, Pux10, and At4g05080, we found that they are strongly downregulated in the mutant *atnp*01, but strongly upregulated in *atnp*02 (Figure 2). This behavior indicates that this group of proteins is involved in an opposite manner within the response pathways that the two different mutants display towards the treatment. In the upper part of the heatmap in Figure 2, it is possible to observe several strongly upregulated proteins only in the mutant *atnp*02. These proteins are: Hsp70-10, a mitochondrial heat shock protein involved in the response to stress, Cat2, catalase 2, involved in the detoxification of hydrogen peroxide, Clpc2, a chloroplast chaperon protein with ATPase activity, Gldp2, a glycine dehydrogenase involved in the degradation of glycine in the mitochondrion, Cul3-B, cullin 3B, active in protein ubiquitination, and Atk5, kinesin 5, with microtubule-binding activity in the cytoskeletron. These proteins reflect the activity of the plant to control the oxidative stress and damage to mitochondria brought about by the CdS QDs.

**Figure 3:**
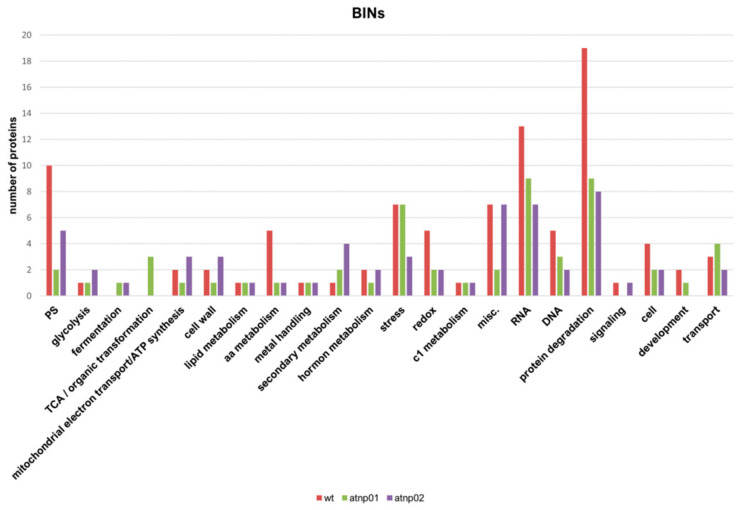
The distribution of differentially abundant proteins in wt, atnp01 and atnp02 according to MapMan ontology classification (BINs). The horizontal axis shows the different MapMan bin types, with the number of proteins for each bin on the vertical axis.

In wt, the set of reprogrammed proteins was associated with the following major MapMan bins: protein synthesis, protein degradation and protein post-transcriptional modification; RNA regulation of transcription; DNA synthesis and chromatin structure; amino acid metabolism; hormone metabolism; redox ascorbate and glutathione biosynthesis; photosynthesis and photorespiration and abiotic and biotic stresses (Figure 3). In *atnp*01, the set of reprogrammed proteins was associated with protein degradation, RNA regulation of transcription, abiotic and biotic stress, and mitochondrial electron transport and ATP synthesis (Figure 3). In *atnp*02, the set of reprogrammed proteins was associated with protein folding, protein degradation, RNA regulation of transcription, photosynthesis (PS), secondary metabolism, mitochondrial electron transport/ATP synthesis, hormone metabolism, abiotic and biotic stress (Figure 3).

### 3.2. Ontology Analysis of the Identified Proteins

**Figure 4:**
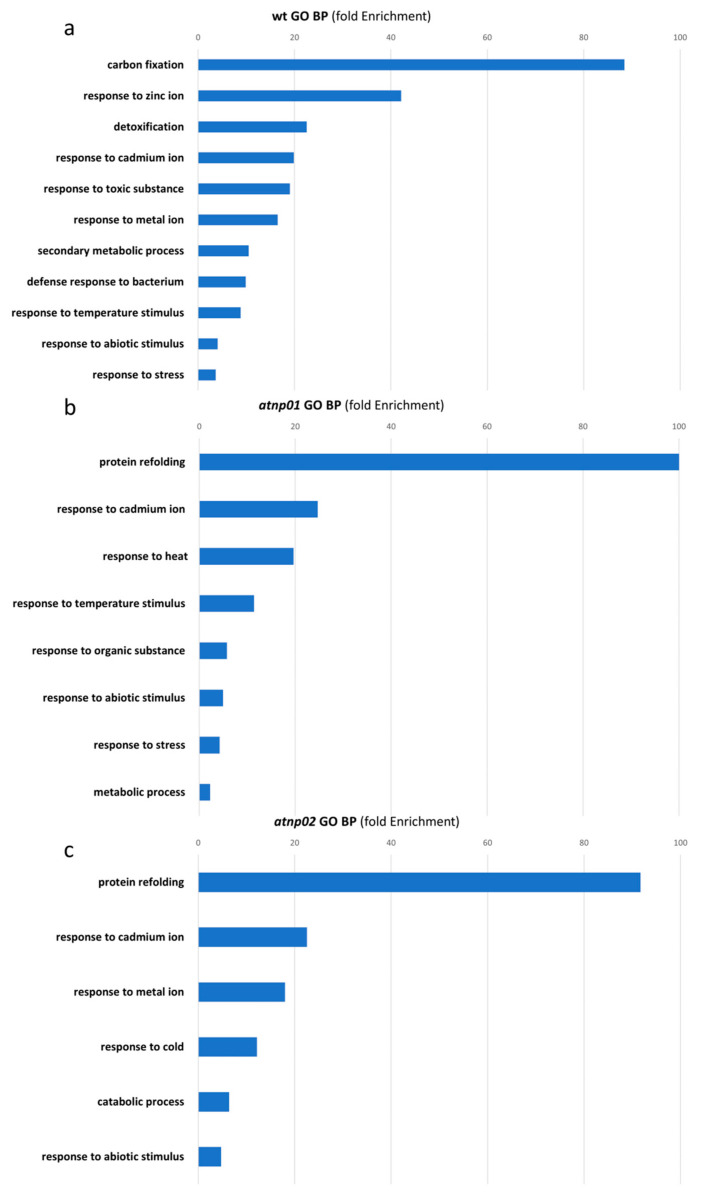
Gene ontology and analyses with n-fold enrichment = −log10 (Fisher’s exact *p*-value) for Biological process in wt (**a**), *atnp*01 (**b**) and *atnp*02 (**c**).

We classified each protein into one of three ontology categories: biological processes, molecular function, and cell components. For the proteins significant when comparing treated wt vs. control wt, the biological processes that were found more enriched were: carbon fixation, response to zinc ion, detoxification, response to cadmium ion, response to abiotic stimulus, and response to stress (Figure 4A). Molecular function categories enriched for this comparison were: glutathione binding, oligopeptide binding, and glutathione transferase activity, while the cell compartments were: apoplast, plastoglobule, chloroplast stroma, thylakoid, photosynthetic membrane, cell wall, organelle envelope, cytosol, and plastid (Figure S5A). The biological processes showed a strong response to stress in general and to metals in particular, including cadmium, which is a component of CdS QDs. In the enriched molecular functions, we found a predominant role of glutathione, which suggests a general response to oxidative stress caused by CdS QDs exposure [24]. The cell compartment pinpoints the role of the chloroplast in the response to CdS QDs, as already found by Pagano et al. [16]. (Figure S5B). This is in keeping with the oxidative stress response prominent in molecular function, as found by Ruotolo et al. [15]. For the protein differences emerging in the comparison of treated *atnp*01 vs. control *atnp*01, the biological processes that were found most enriched were: protein refolding, response to cadmium ion, response to heat and temperature and response to stress (Figure 4B). For this pair, the enriched molecular function categories were: misfolded protein binding, protein folding chaperone and heat shock protein binding, while the cell components were: endoplasmic reticulum chaperon complex, secretory vesicle, apoplast, cell wall, and cytosol (Figure S5C). Here, it is possible to recognize in the biological process not only responses to abiotic stress and metals, but also a protein folding response which is again predominant in the molecular function. Therefore, it can be summarized that treatment of the mutant line *atnp*01 potentiated several proteolytic mechanisms. Thus, the cellular components affected by the treatment were the endoplasmic reticulum and cell wall where proteolysis is carried out by the cell (Figure S5D). For the proteins emerging in the comparison of treated *atnp*02 vs. control *atnp*02, the biological processes that were found more enriched were: protein refolding, response to cadmium ion, response to metal ion, response to catabolic process and response to abiotic stimulus (Figure 4C). Laware and colleagues [49] found that in seedlings of *Alium cepa* treated with TiO_2_ nanoparticles there was a strong dose-dependent proteolitic activity. For the molecular function of the same comparison the enriched categories were: misfolded protein binding, protein folding chaperone, heat shock protein binding, and nucleoside-triphosphate activity (Figure S5E), while the cell components were: apoplast, plastid envelope, thylakoid, chloroplast envelope and stroma, vacuole, bounding membrane of organelles and cell-cell junction (Figure S5F). Treated *atnp*02 responded in the biological function category with a general stress response and one category of protein folding which is similar to the response of the *atnp*01. Similarly, in terms of molecular function the categories were predominantly related to protein readjustment. In this case, the chloroplast (Gapa1 and Gapa2) and the vacuole (Vps24-1) were involved in the treatment response, which indicates the presence of oxidative stress [16].

### 3.3. Pathways Analysis in Response to CdS QDs Treatment

**Figure 5:**
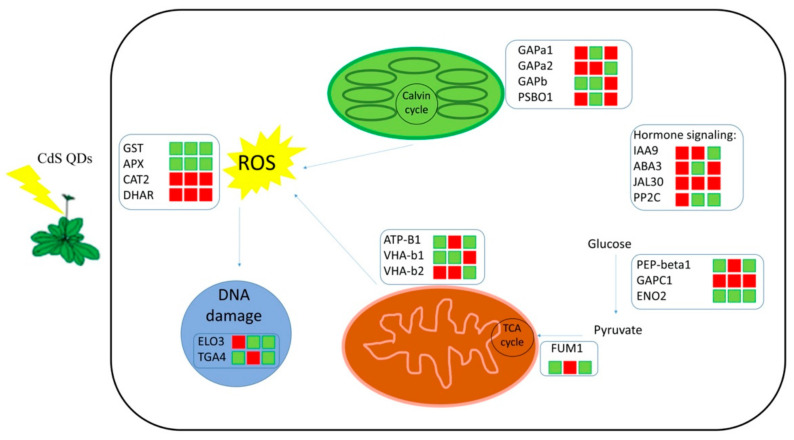
Model depicting the main A. thaliana proteome pathways responsive to CdS QDs treatment. For each protein the colored bars indicate up- (red) or downregulation (green). The first column indicates the up- and downregulated proteins in wt, while the second and third columns similarly show those in mutants *atnp*01 and *atnp*02.

Several pathways were identified by Mapman ontology as being particularly involved in the response to CdS QDs. In wt and both mutants, proteins from cellular metabolism such as glyceraldehyde-3-phosphate dehydrogenase (Gapc1, Gapb, Gapa1 and Gapa2), pyrophosphate fructose6-phosph1-phosphotransferase subunit beta1 (Pep-beta1), 30S ribosomal protein S5, glycine dehydrogenase decarboxylating 2 (Glpd2) and bifunctional enolase 2/transcriptional activator (Eno2) were modulated (Figure 2 and Figure 3 and Table S2). Arabidopsis has four different GAPDH isoforms, with seven phosphorylating types and one non-phosphorylating. These include cytosolic glycolytic GAPDHs (GAPC1 and GAPC2), chloroplastic photosynthetic GAPDHs (GAPA1, GAPA2, and GAPB), plastidic glycolytic GAPDHs (GAPCp1 and GAPCp2), and the NADP-dependent non-phosphorylating cytosolic GAPDH (NP-GAPDH) [50]. Substrate conversion by glycolytic GAPDHs catalyzes a simultaneous reduction of NAD^+^ to NADH [51]. Arabidopsis GAPA1/2 and GAPB use NADPH to generate NADP^+^, which buffers free radical generation from the electron transport chain by dissipating the H+ gradient in the thylakoid membrane [52,53]. Therefore, by contributing to the maintenance of the NAD(P)^+^/NAD(P)H ratio of the cell, plant GAPDHs influence cellular redox and general metabolism. In particular, Gapc1 plays a role in the glycolytic pathway, but at the same time, it can interact with H_2_O_2_ thus becoming part of the ROS signaling cascade [51]. The regulation of several proteins involved in primary metabolism suggests that CdS QDs exposure had moderately influenced carbon metabolism. These include two proteins involved in the glycolytic pathway: the upregulation in wt of Gapc1 and the downregulation in two mutants of Eno2 (Figure 2, Figure 3 and Figure 5 and Table S2). This observation further supports the concept that CdS QDs exerts oxidative stress by reacting with cellular proteins and enzymes and subsequently generated free radicals [54]. As hypothesized by Tiwari et al. [55], the disruption or malfunction of the electron transport system in mitochondria and chloroplast by QDs, once they are inside the cell, could lead to ROS production [55]. In wt and the two mutants many proteins are involved in mitochondrial electron transport including V-type proton ATPase subunit B1 and B2 (Vha-b1, Vha-b2), prohibitin-3 (Phb3), and gamma carbonic anhydrase 2 (Gammaca2), with, in the chloroplast Calvin cycle, phosphoribulokinase (At1g32060) and chaperonin 60 subunit beta 1 (Cpn60b1) (Figure 3 and Table S2). It has been demonstrated that physical interaction with ENMs disrupts the normal function of organelles in cells. For example, mitochondrial damage is thought to be one of the possible mechanisms of ENMs cytotoxicity by inducing oxidative stress through the destruction and redistribution of normal electron transport by respiratory complexes [56]. V-type proton ATPase subunit B1 was downregulated in wt and in *atnp*01, while V-type proton ATPase subunit B2 was downregulated in *atnp*02 (Figure 2 and Figure 5). A study on Arabidopsis mitochondrial proteomics identified enolase among the enzymes associated with the outer mitochondrial membrane [57], and enolase is also proven to interact with the tonoplast through direct association with V-ATPase subunits, specifically the regulatory subunit Vha-b [58]. According to Ruotolo et al. [15], CdS QD treatment decreases respiratory efficiency and chlorophyll content in Arabidopsis [15]. Therefore, our findings support the involvement of the mitochondria respiratory process in CdS QDs responses in wt and both mutants. Interestingly, in the two mutants, respiration and photosynthesis were less sensitive to QDs than wt. Most of the studies carried out so far have dealt primarily with the overall plant stress response towards specific groups of ENMs, showing a differential abundance of proteins involved in oxidation-reduction, reactive oxygen species (ROS) detoxification, stress signaling, and hormonal pathways [59].

Interestingly, five glutathione S-transferases (GSTs), representative of λ and ϕ classes of the GST family, have been identified in the proteome analysis (Figure 2 and Table S2). In wt, two GST family members, Gst F2 and F9, decreased under treatment, while GstF7 and Gst-darh3 increased; conversely, in *atnp*02, GstF3 was upregulated (Figure 2 and Figure 5). The GST family in plants is notable for its structural and functional diversity, but the biochemical and physiological functions of each specific member remain to be clarified. As well as, or instead of, catalyzing conjugase reactions, some GSTs have antioxidative functions. The DHAR type of GST is one example. In addition, several subclasses of GST have peroxidase activity [60,61]. The detection of a large number of GSTs in the A. thaliana proteome is quite interesting; the functions of GSHs in the maintenance of the cell’s redox balance, in xenobiotic detoxification and flavonoids subcellular transport is well known. GST expression is induced by a broad swathe of stressing conditions [62], including ENMs [63,64]. In Arabidopsis, ENMs induce an oxidative stress response by producing ROS, as has also been reported in crop plants [64,65]. Genes encoding for proteins of the NADPH oxidase and superoxide dismutase (SODs) families, and particularly peroxidases (PODs) and gluthatione S-transferase (GST) families, involved in antioxidant pathways that promote ROS detoxification, become significantly modulated under CdS QD and CuO and ZnO NP treatments [24,66,67].

Exposure to ENMs may result in the change of transcription of genes involved in hormone signaling pathways, e.g., of auxin repressor or auxin response genes, abscisic acid (ABA) biosynthetic genes, or ethylene signaling components [68]. The present study, shows that in A. thaliana wt CdS QDs caused upregulation of the protein involved in the biosynthesis of auxins (auxin-responsive protein IAA9); in *atnp*01 there was downregulation of proteins involved in the biosynthesis of ABA (molybdenum cofactor sulfurase), and in *atnp*02 there was upregulation of a protein involved in the biosynthesis of jasmonate (JA) (PYK10-binding protein 1) and downregulation of the protein involved in the biosynthesis of auxin (IAA9) (Figure 2 and Figure 5 and Table S2). ABA plays a key role in lateral root formation inhibition in plants exposed to environmental stress [69], and it is also an antagonist of brassicosteroids-promoted growth. Genes induced by ABA are upregulated by CdS QDs [14], CuO [66], and ZnO [67] NPs. In plants, protein phosphatase 2C 58 (PP2C) has been connected with the negative regulation of protein kinase cascades that are activated because of stress. Members of the PP2C family, such as ABI1 and ABI2, are involved in ABA signal transduction. ABA is a plant hormone crucial in mediating the plant responses to environmental stresses [70]. In this work, we found that ABI1 and ABI2 are upregulated in the wt and downregulated in both mutants when treated with CdS QDs (Figure 2 and Figure 5). These proteins are downregulated in both mutants probably because the signal transduction carried out by the hormone ABA is impaired in both mutants by the nanoparticles. The major role of JA (jasmonic acid) is defense against pathogens. However, this hormone plays a role in plant growth control [71]; the transcription of some JA responsive genes increased upon exposure to certain ENMs. Several genes involved in SA (salicilic acid) pathways, a signaling molecule that is important in general plant stress response, were downregulated by early exposure to Ag and TiO_2_; however, they were upregulated by exposure to CdS QD, CuO, and ZnO NPs [14,66,67]. The modulation of these proteins by CdS QDs treatment highlights the importance of hormones and signaling in the response mechanisms to these nanoparticles. Figure 5 gives an overview of all the above-mentioned pathways responsive to treatment with CdS QDs.

### 3.4. Network Analysis

**Figure 6:**
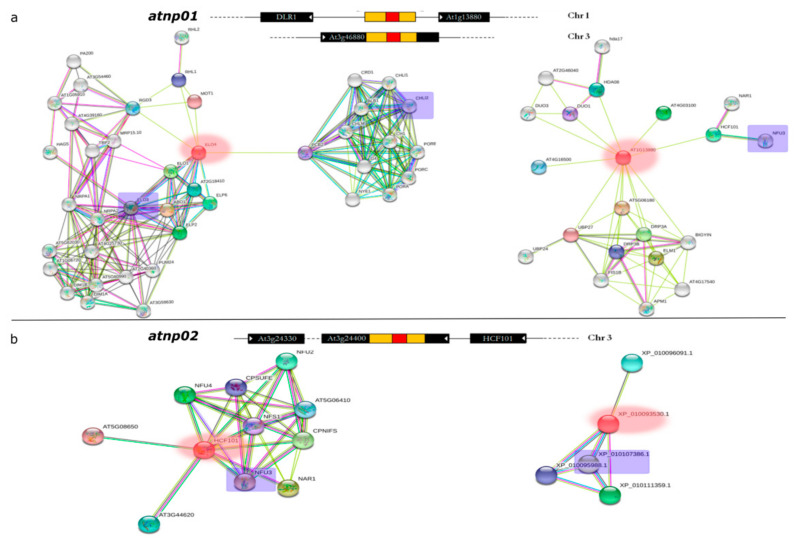
Molecular interaction network of differentially abundant proteins. The mutated genes in the two mutant lines are shown in red in all the networks. A schematic above each network shows the structure of the mutated genes as in [14]. The segment in red and yellow is the insertion element (Ac), the segments in black are the mutated genes and Chr indicates the chromosomes on which these genes are positioned. The putative proteins that were found in the protein analysis and that connect in the network are indicated with blue rectangles. Networks for mutant *atnp*01 (**a**) and mutant *atnp*02 (**b**). Colored lines and dots indicate different types of interaction evidence (cyan, from curated databases; green, gene neighborhood; blue, gene co-occurrence; pink, experimentally determined; black, co-expression; light blue, protein homology).

Starting from the molecular structure of the two mutants *atnp*01 and *atnp*02, and using a network analysis, we tried to find any among the identified proteins which showed variations that could be correlated with the mutations. Three candidate genes were potentially affected by the mutagenic events in *atnp*01 [14]. Two of these genes (At1g13880 and DLR1) are positioned on chromosome 1, the other (At3g46880) is on chromosome 3 (Figure 6) and encodes an unknown protein localized in the chloroplast, which does not interact with any other protein. DRL1 encodes a cytoplasm-localized calmodulin binding protein involved in leaf development and meristem structural organization. DLR1 is a homolog of the yeast TOT4/KTI12 protein which associates with elongator, a multisubunit complex binding to RNA polymerase II transcription elongation complex [72]. DRL1 interacts with two differentially abundant proteins found with 2D-PAGE: elongator complex protein 3 (Elo3) and magnesium-chelatase subunit ChlI-2 (Chli2) (Figure 6A), which are respectively down- and upregulated in atnp01. Elo3 is one of the six subunits of Arabidopsis Elongator Complex (AtELP) [73]. Atelp/elo mutants display pleiotropic phenotypes, including resistance to oxidative stress, hypersensitivity to abscisic acid, severely aberrant auxin phenotypes, altered cell cycle progression, abnormal root development, and disease susceptibility [74,75,76]. Recent studies have shown that AtELP3, together with AtELP2, regulates the kinetics of pathogen-induced transcriptome reprogramming [77]. CHLI isoforms in Arabidopsis are encoded by two genes: Chli1 (At4g18480) and Chli2 (At5g45930). Chli1 seems to be the major functional form since chlorophyll levels in chli1-null mutants are reduced to 10% to 17% of the wild-type level [78]. It has been hypothesized that Chli2 could support some chlorophyll biosynthesis in the complete absence of Chli1 [79]. At1g13880 (Elm2) is a member of the MYB class of transcription factors. Elm2 has only a limited role in mitochondrial fission, but it is a 54% paralog with ELM1 which has a fundamental function in mitochondrial fission [80]. Elm2 interacts with one differentially abundant upregulated protein, NifU-like protein 3 (Nfu3), which is involved [81] in the cluster assembly of chloroplastic Fe-S proteins (Figure 6A).

The *atnp*02 mutagenic event affected genes on chromosome 3, potentially At3g24330, encoding an O-glycosyl hydrolase localized in the endomembrane, and At3g24430 (Hcf101) encoding an ATP binding protein localized in the chloroplast. The mutagenic element lay within the At3g24400 pseudogene (AtPERK2), which possibly encodes a proline-rich extensin-like receptor kinase [14]. Hcf101 results were bound in the network with Nfu3, which in this case is downregulated (Figure 6B). The gene Perk2 interacts with the downregulated protein phosphatase 2C (At4g28400) (Figure 6B). This protein has already been described in Section 3.1 and Section 3.3 and is the only one in common among the wt and the two mutants. The hypothesis is that the proteins related to these mutated genes were also the main targets in the signal cascade deriving from interaction with CdS QDs during the treatment.

### 3.5. Comparison between 2D-PAGE, Pf2D, and Transcriptomics

Marmiroli et al. [25] compared the proteomes of wt and the two mutants, treated with 80 mg/L CdS QDs, using a gel-less technique based on liquid IEF in the first dimension and HPLC in the second dimension, performed with a ProteomeLab^TM^ PF2D (Beckman Coulter) [25]. The IEF gradient was pH 4–8.5 and the most water-soluble proteins were separated with this method. In our experiments we found four proteins in common with Marmiroli et al. [25]. For the wild type: ribulose bisphosphate carboxylase large chain (rbcL) (Rubisco large chain) [82], (downregulated in both cases); glutathione S-transferase DHAR3 (Dhar3) and pathogenesis-related protein 5 (At1g75040) (both proteins upregulated in all cases); for the mutant *atnp*02: heat shock 70 kDa protein 10 mitochondrial (Hsp70-10) (upregulated). For the mutant *atnp*01 there were no common proteins.

Plant pathogenesis-related (PR) proteins are expressed in response to pathogen attack, developmental processes and environmental stresses; some of these proteins are constitutively expressed [83]. Hsp70-10 was required for transport of secretory proteins from the Golgi complex [84]. Comparing our data with the transcriptomics results reported in Marmiroli et al. [14], we found three proteins in common: osmotin-like protein OSM34 (Osm34), pathogenesis-related protein 5 (At1g75040) and phosphatidylinositol/ phosphatidylcholine transfer protein SFH3 (Sfh3). OSM34 was always upregulated both in the transcriptomics and in the proteomics in the wt and in the two mutants. This protein is usually considered a response protein to osmotic stress [85]. Pathogenesis-related protein 5 was upregulated in the transcriptomics, and in the proteomics was also upregulated in both wt and mutant *atnp*01. This protein was in common with the gel-less proteomic study performed with PF2D (see above). For all lines Sfh3 was found downregulated in the transcriptomic, while in our study was upregulated in *atnp*01 and downregulated in wt and in *atnp*02. These common proteins are mostly related to the oxidative stress response, which seems to be the driving stress arising from the interactions with CdS QDs [15].

**Figure 7:**
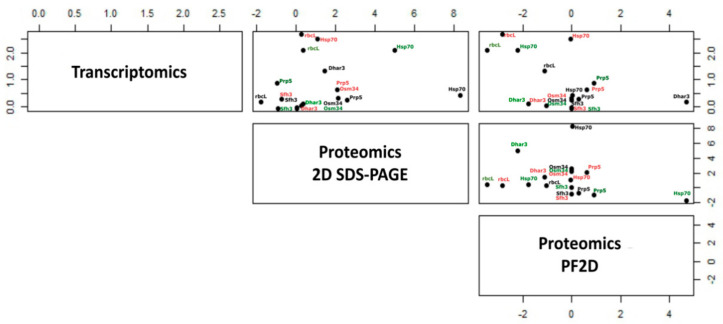
Scatterplot matrix of the independent variables: transcriptome, proteome with 2D-PAGE, and proteome with PF2D. The three-dimensional scatterplot represents the correlation among gene expression, protein abundance with 2D-PAGE and protein abundance with Pf2D. The lines are represented in black for wt, in red for *atnp*01, and in green for *atnp*02. Transcriptomics data are taken from array analysis in Marmiroli et al. [14], and proteomic with PF2D data from Marmiroli et al. [25]. Each point represents a protein/transcript for the different plant lines using colors as above.

Figure 7 shows the levels of correlation between proteomics (2D-PAGE)/transcriptomics, proteomics (PF2D)/transcriptomics, and proteomics (2D-PAGE)/proteomic (PF2D) markers. The figure was obtained by comparing 98 significant proteins obtained with 2D-PAGE with 88 selected proteins obtained with PF2D and the significant group of transcripts obtained with an Arabidopsis microarray platform. It is well known that the correlation between proteomics and transcriptomics is moderate to low [25,29], and in this case is also strongly biased by the difference between the higher number of transcripts and the lower number of proteins. Therefore, the proteins/transcripts considered as molecular markers showing this degree of correlation in the three comparisons are viewed as robust enough to be considered candidate “-omics” CdS QDs exposure markers.

## 4. Conclusions

The majority of the differential abundance proteins in the wt were downregulated on exposure to CdS QDs and in Mapman bins annotated to processes like protein folding, biotic and abiotic stress responses, and protein degradation. Conversely, in both mutants, there was a balance in numbers between reprogrammed up- and downregulated proteins (Figure 2). Mapman bins for the mutant *atnp01* were protein degradation, abiotic stress and mitochondrial electron transport. These pathways are all typical of the responses to nanomaterials [86]. For the mutant *atnp02*, the main Mapman bins were glycolysis, mitochondrial electron transport, photosynthesis and hormone metabolism [15,86]. These results indicate that in *atnp02* the photosynthetic apparatus was impaired by treatment with CdS QDs, in addition to the other categories, especially hormone metabolism and mitochondrial electron transport chain, which were common to both mutants.

Network analysis showed that in the two mutants, the genes affected by the transposons are responsible for regulation of four proteins: *Nfu, Elo3, Chli2* and *PP2c*, involved respectively in chloroplast assembly, transcription elongation, chlorophyll biosynthesis and abiotic stress response. The reprogramming of these particular proteins demonstrates the importance of the chloroplast and of photosynthesis in the responses to CdS QDs of the mutants.

The authors are perfectly aware that there are more powerful proteomic tools available for plants (i.e., iTRAQ) which can give higher resolution and better quantification than 2D-PAGE. The purpose of this paper was to compare protein variations resulting from samples treated with CdS QDs vs. untreated controls, which certainly may benefit from powerful proteomic tools, but at the same time to probe these variations with the aid of a genetic tool, two CdS QDs-tolerant mutants. The use of the two mutants allowed refining the protein comparison from a large systematic and almost taxonomic level to a comparison in which a small proportion of proteins were objectively important. The two mutants consistently narrowed the proteomic range, allowing focus on those proteins specific for the tolerant phenotype rather than attention being dispersed on a plethora of proteins that were more aligned to networking effects. This approach is suitable not only following treatment with CdS QDs, but also for many other stressed conditions.

## Data Availability

Publicly available datasets were analyzed in this study. This data can be found here: [doi:10.1021/es404958r; doi.org/10.3389/fpls.2015.01104].

## Supplementary Materials

The following are available online at https://www.mdpi.com/2079-4991/11/3/615/s1, Supplementary File 1: S.1. Supplementary methods related to nanoparticles synthesis and characterization. S.2. MapMan pathways identified under QDs treatment. Figure S1. HRTEM image of ligand-free QDs assembly and X-ray diffraction pattern. Figure S2. (A) ESEM image of the CdS QDs agglomerates. (B) X ray spectra corresponding to the red rectangle in figure S2A. Figure S3. 2D SDS-PAGE. Figure S4. Venn diagrams for common and non-common proteins to wt and *atnp01*, to wt and *atnp02*, to *atnp01* and *atnp02* (a) in the control and (b) in treatment conditions. Figure S5. Heat map of *A. thaliana* wt and mutant lines *atnp01* and *atnp02* not treated and treated with 80 mg L^−1^ CdS QDs. Figure S6. Gene Ontology and enrichment analyses with fold enrichment = −log10 (Fisher’s exact *p* value) for molecular function wt (a); cellular component wt (b); molecular function *atnp01* (c); cellular component *atnp01* (d); molecular function *atnp02* (e), cellular component *atnp02* (f). Figure S7. Cell function overview map after CdS QDs exposure. Cell functions associated with the proteomic changes affecting *Arabidopsis thaliana* after CdS QDs exposure in wt (a) *atnp01* (b) and in *atnp02* (c) using MapMan software. Figure S8. Biotic stress overview map after CdS QDs exposure. Stress response associated with the proteomic changes affecting A. thaliana after CdS QDs exposure in wt (a) *atnp01* (b) and in *atnp02* (c) using MapMan software. Table S2. MapMan BIN assignation and description of differentially expressed proteins in *Arabidopsis thaliana.* Supplementary File 2: Table S1. MALDI-TOF/TOF data associated with differentially expressed proteins identified by 2D-PAGE.
