# Supplementary material for: Comparative Analysis of Proteins Regulated during Cadmium Sulfide Quantum Dots Response in Arabidopsis thaliana Wild Type and Tolerant Mutants
Source: Nanomaterials (Basel). 2021 Mar 1;11(3):615. doi: 10.3390/nano11030615 (PMC7998754; doi:10.3390/nano11030615)
Supplement: Supplementary file 1 [file nanomaterials-11-00615-s001.zip › Supplementary.docx]

**Supplementary file 1.**

**Comparative analysis of proteins regulated during cadmium sulphide quantum dots response in *Arabidopsis thaliana* wild type and tolerant mutants**

*Valentina Gallo^1^, Andrea Zappettini^2^, Marco Villani^2^, Nelson Marmiroli ^1,3^, Marta Marmiroli ^1 *^.*

^1^ Department of Chemistry, Life Sciences and Environmental Sustainability, University of Parma, 4 Parma, Italy

^2^ Institute of Materials for Electronics and Magnetism (IMEM), National Research Council (CNR), 6 Parma, Italy

^3^ The Italian National Interuniversity Consortium for Environmental Sciences (CINSA), Parma, Italy

**List of Supplementary material:**

S.1 Supplementary methods related to nanoparticles synthesis and characterization

S.2 MapMan pathways identified under QDs treatment

Figure S1. HRTEM image of ligand-free QDs assembly and X-ray diffraction pattern

Figure S2. (A) ESEM image of the CdS QDs agglomerates. (B) X ray spectra corresponding to the red rectangle in figure S2A.

Figure S3. 2D SDS-PAGE.

Figure S4. Venn diagrams for common and non-common proteins to wt and *atnp01*, to wt and *atnp02*, to a*tnp01* and *atnp02* (a) in the control and (b) in treatment conditions.

Figure S5. Heat map of A. thaliana wt and mutant lines atnp01 and atnp02 not treated and treated with 80 mg L^-1^ CdS QDs.

Figure S6. Gene Ontology and enrichment analyses with fold enrichment = -log10 (Fisher’s exact p value) for A) Molecular function wt; B) cellular component wt; C) molecular function *atnp01,* D) cellular component *atnp01*; E) molecular function *atnp02,* F) cellular component *atnp02.*

Figure S7. Cell function overview map after CdS QDs exposure. Cell functions associated with the proteomic changes affecting *Arabidopsis thaliana* after CdS QDs exposure in wt (A) *atnp01* (B) and in *atnp02* (C) using MapMan software. The represented squares are only for proteins showing a significant change in protein abundance between the treatment and the untreated control that were attributed to the respective bins by MapMan. Over- and under- abundant proteins are indicated in red and green, respectively.

Figure S8. Biotic stress overview map after CdS QDs exposure. Stress response associated with the proteomic changes affecting *A. thaliana* after CdS QDs exposure in wt (A) atnp01 (B) and in atnp02 (C) using MapMan software. The represented squares are only for proteins showing a significant change in protein abundance between the treatment and the untreated control that were attributed to the respective bins by MapMan. Over- and under- abundant proteins are indicated in red and green, respectively.

Table S2. MapMan BIN assignation and description of differentially abundant proteins in *Arabidopsis thaliana.*

*S.1. CdS QDs synthesis and characterization*

Uncoated Cadmium Sulfide Quantum Dots (CdS QDs) were synthesized by IMEM-CNR (Parma, Italy), following the method of Villani *et al*. (2012) [1]. The CdS QDs were characterized in deionized water by transmission electron microscopy (TEM) (Hitachi HT7700, Hitachi High Technologies America, Pleasanton, CA) [2]. Average static diameter was 5 nm, and the crystal structure was that of wurtzite (ZnS) with approximately 78% Cd. Average particle size (dh) of the aggregates (measured with DLS) and zeta potential (ζ) in ddH_2_O were estimated in deionized water at 178.7 nm and +15.0 mV, respectively (Zetasizer Nano Series ZS90, Malvern Instruments, Malvern, UK). An ESEM Quanta 250FEG, FEI with Bruker QUANTAX EDS XFlash® 6T detector series and ESPRIT 2 analytical methods interface (FEI company, 5350 NE Dawson Creek Drive Hillsboro, Oregon 97124 USA, Bruker, Am Studio 2D, 12489 Berlin, Germany) was utilized to determine CdS quantum dots group morphology and elemental content. Single drops of 1 ml containing 80 mg/l of CdS Quantum dots were left to dry on SEM stub covered with carbon tape in a protected environment. Seven stubs were analyzed during one round of experiments. Working parameters for SE imaging e X-ray spectra acquisition were as follows. Pressure: 70 Pa, working distance: 9.9 mm, acceleration voltage: 20KeV. Figure S2A, B represents CdS QDs at different magnifications, along with their EDX spectra [2]. Nanoarticle characterization data and images are provided in Figures S1-S2.

*S.2 MapMan pathways identified under QDs treatment*

Metabolic pathways associated with the cellular status of *Arabidopsis thaliana* wt and both mutants were derived using MapMan software, based on the “Ath_AGI_TAIR9_Jan2010” database. The highest scoring processes were “cell function overview” and “biotic stress pathway” for the three groups of genetic backgrounds. The “cell function overview” assigned 57 of 61 mapped proteins in the wt to 14 processes, namely: “DNA synthesis”, “cell organization”, “stress biotic and abiotic”, “regulation of transcription”, “development”, “protein synthesis and amino acid activation”, “hormone”, “regulation”, “protein modification”, “protein degradation”, “redox”, “metal handling”, “transport” and “enzyme families”, leaving the remaining four proteins either without any ontology or having an unknown function (Figure S7A). Of the 31 atnp01 proteins classified within the “cell function overview” category, 29 could be assigned to 12 processes “cell division and cell cycle”, DNA synthesis”, “stress biotic and abiotic”, “regulation of transcription”, “hormone”, “protein modification”, “protein degradation”, “redox”, “transport” and “enzyme families”, leaving the remaining 4 proteins either without any ontology or having an unknown function (Figure S7B). Of the 31 atnp02 proteins classified within the “cell function overview” category, 28 could be assigned to 13 processes: “DNA synthesis”, “cell organization”, “stress biotic and abiotic”, “regulation of transcription”, “protein synthesis and amino acid activation”, “hormone”, “regulation”, “protein modification”, “protein degradation”, “redox”, “metal handling”, and “enzyme families”, leaving the remaining 3 proteins either without any ontology or having an unknown function (Figure S7C).

The 23 wt proteins assigned to the “biotic stress pathway” were associated with “hormone signaling”, “cell wall”, “proteolysis”, “heat shock proteins” “redox”, “abiotic stress” “signaling” “transcription factor” and “secondary metabolites” (Figure S8A). The 16 atnp01 proteins assigned to the “biotic stress pathway” were associated with “hormone signaling”, “transcription factor” and “secondary metabolites” (Figure S8B). The 10 atnp02 proteins assigned to the “biotic stress pathway” were associated with “cell wall”, “proteolysis”, “heat shock proteins” “redox” and “abiotic stress” (Figure S8C). Proteins that have been experimentally indicated to be involved in biotic stress are collected in the main panel (colored with dark grey), while proteins and pathways that are putatively involved in biotic stress are shown on sides (colored in light grey).

The MapMan pathway analysis highlighted the distinct behavior adopted by wt in respect to both mutants after CdS QDs exposure. Proteins implicated in the biotic stress response were reprogrammed in wt and mutants, but there were differences with respect to the number and type of the major classes of proteins involved. For example, transcription regulation, protein degradation, and redox were prominent in wt, whereas in both mutants, the main categories were protein degradation, biotic and abiotic stress and hormones.

**References**

1. Villani, M., Calestani, D., Lazzarini, L., Zanotti, L., Mosca R., Zappettini, A. Extended functionality of ZnO nanotetrapods by solution-based coupling with CdS nanoparticles. *J. Mater. Chem.* **2012**, *22*, 5694.
2. Marmiroli, M.; Mussi, F; Pagano, L; Imperiale, D.; Lencioni, G.; Villani, M; Zappettini, A.; White, J. C.; Marmiroli, N. Cadmium sulfide quantum dots impact *Arabidopsis thaliana* physiology and morphology. *Chemosphere* **2020**, *240*, 124856.


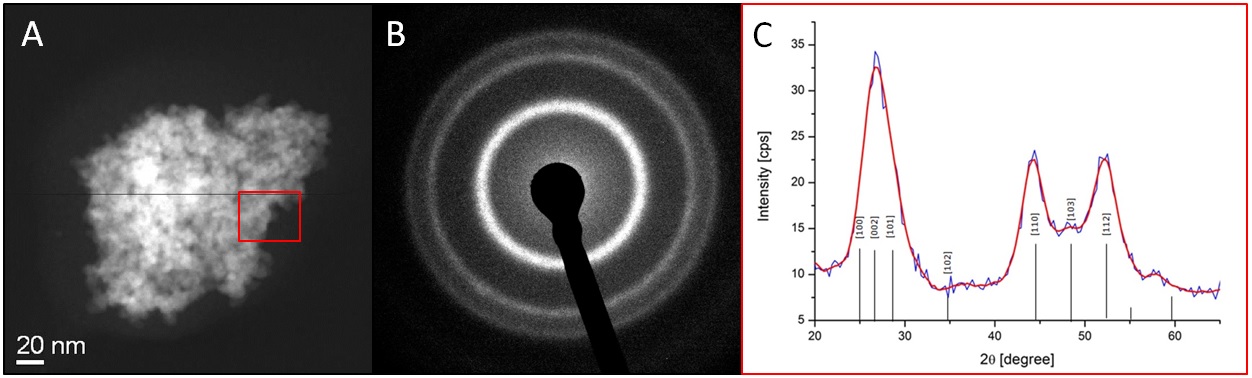


Figure S1. HRTEM image of ligand-free QDs assembly. (A) A CdS QDs aggregate upon solvent evaporation. (B) Fourier transform analysis of the whole HRTEM image. (C) X-ray diffraction pattern. Diffraction pattern obtained from the red rectangle: the diffraction rings related to the lower diffraction index are observable related to the hexagonal structure of the CdS QDs. From inside to outside are observable the triplet 100,002, 101; the doublet 110 e 103; and the singlet112. According to the diffraction pattern the estimated dimensions of the QDs are between 4 and 5 nm.


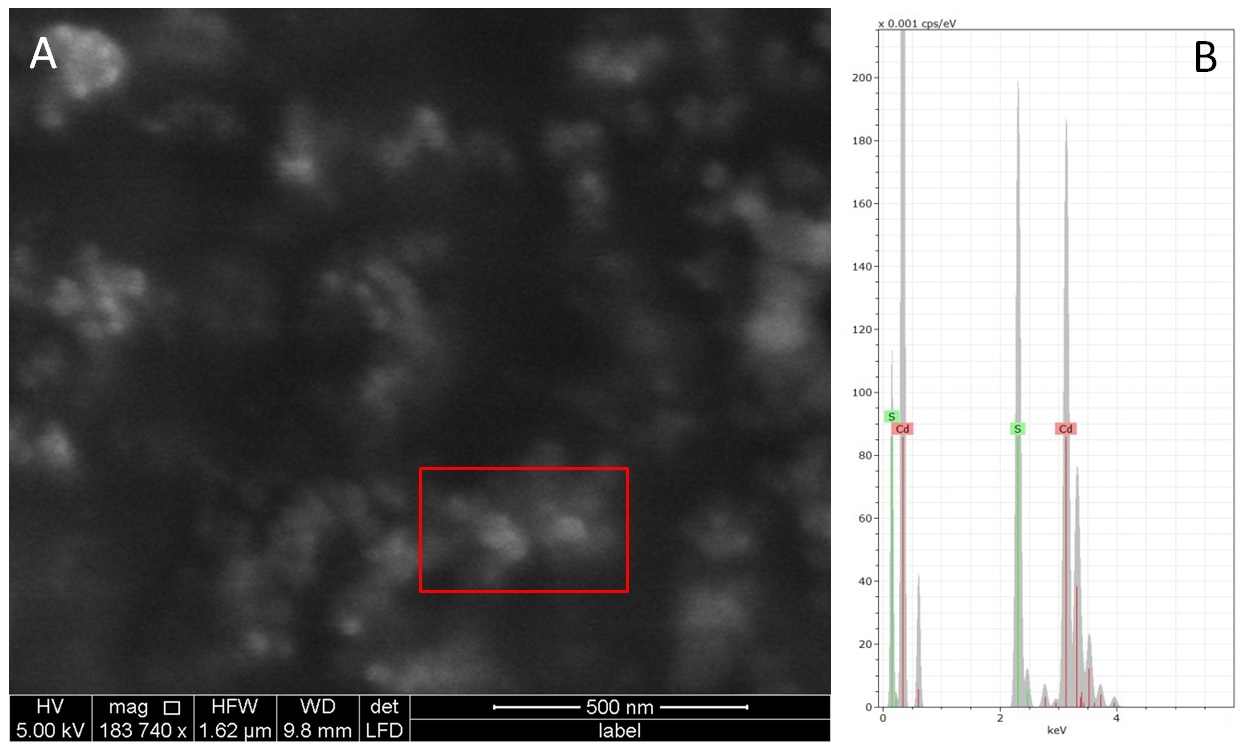


Figure S2. (A) ESEM image of the CdS QDs agglomerates. (B) X ray spectra corresponding to the red rectangle in figure S2A.


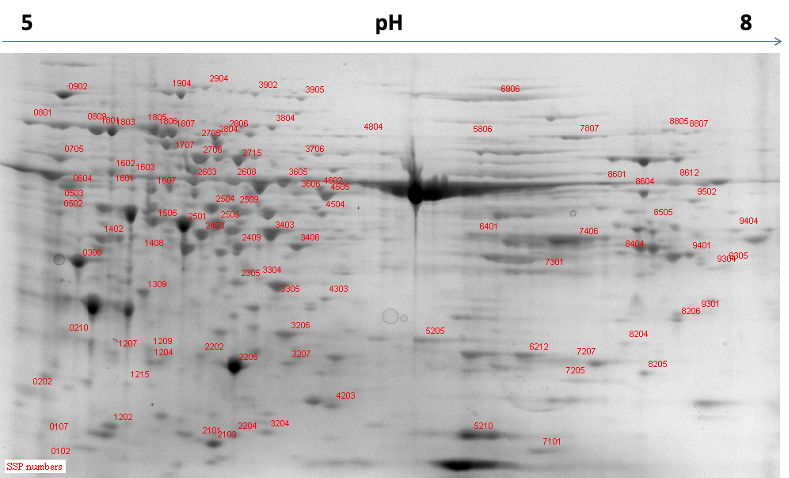


Figure S3. One of the 2D SDS-PAGE representative gel. *Arabidopsis thaliana* proteins (400 μg loaded) were separated over pH range 5-8 (11 cm strips) and 12% SDS-polyacrylamide gel. This gel was stained with Comassie Brillant Blue. Red numbers indicate proteins showing quantitative differences, which were further identified by MS approach.


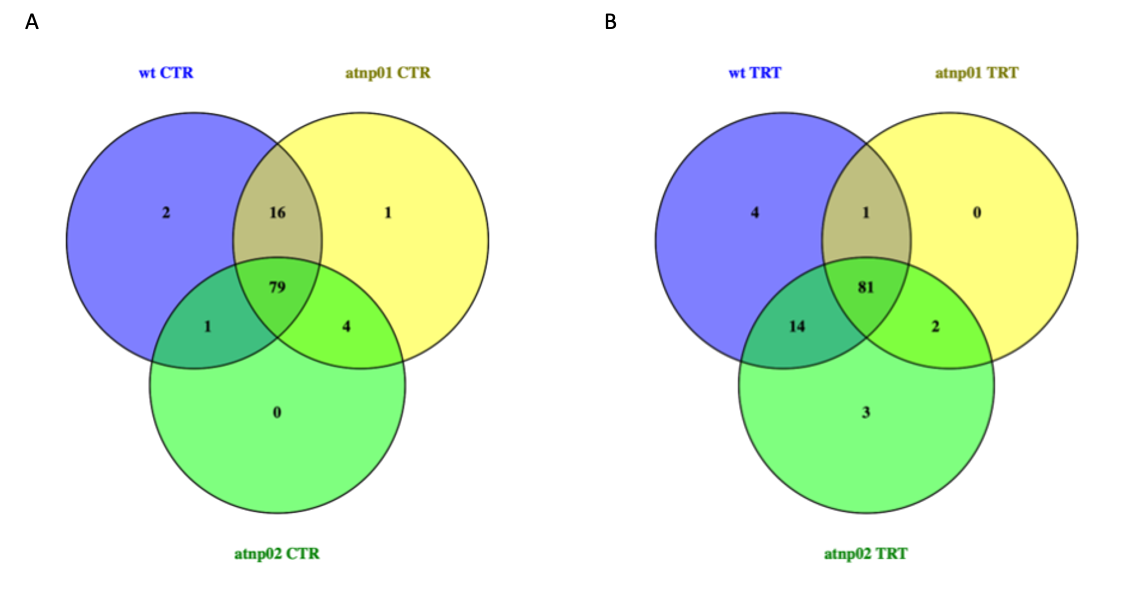


Figure S4: Venn diagrams for common and non-common proteins to wt and *atnp01*, to wt and *atnp02*, to a*tnp01* and *atnp02* **(a)** in the control the common proteins between wt and *atnp01* are 95, the common proteins between wt and *atnp02* are 80, the common proteins between the two mutants are 83; **(b)** In treatment conditions the common proteins between wt and *atnp01* are 82, between wt and *atnp02* are 95, between the two mutants are 83.


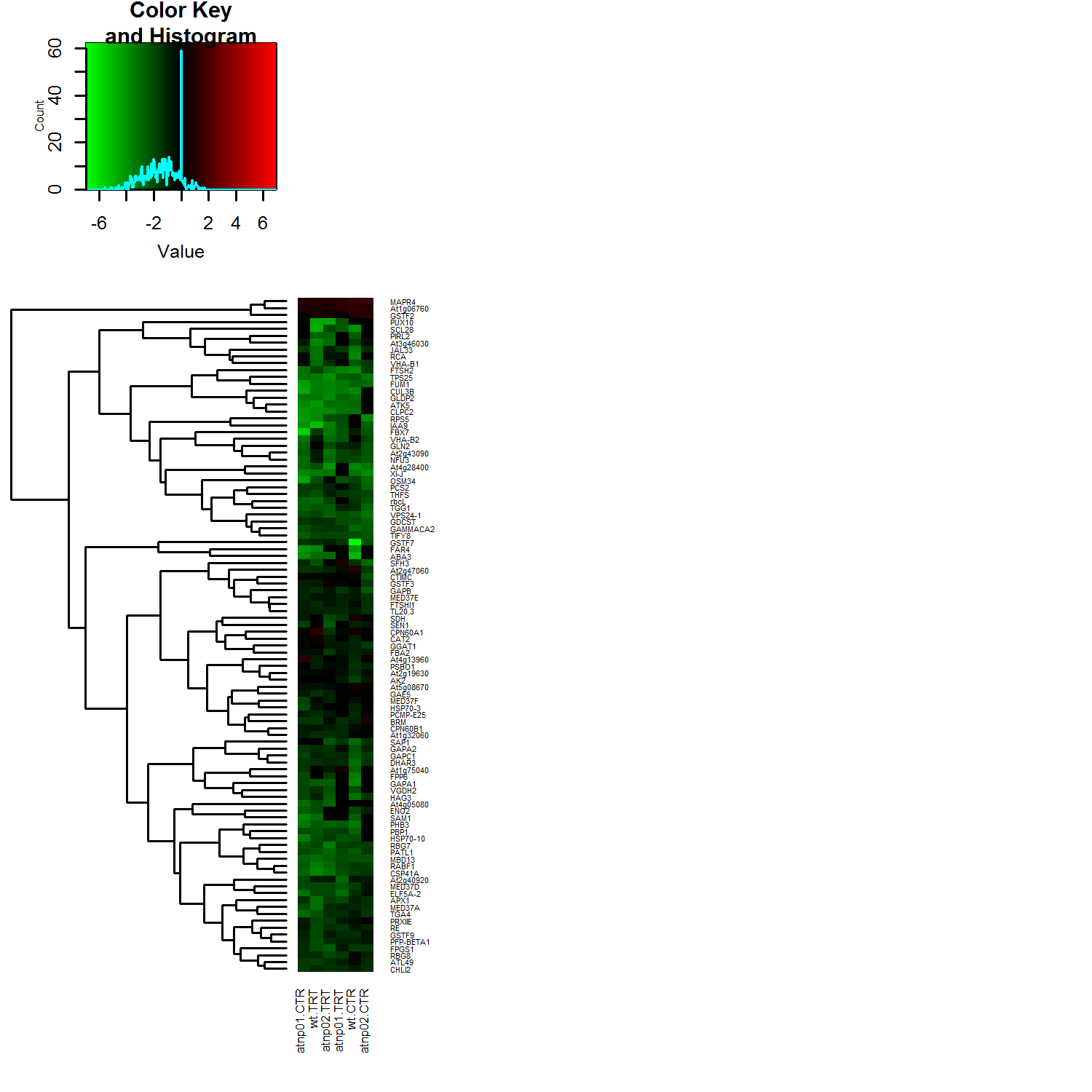


Figure S5. Heat map of *A. thaliana* wt and mutant lines *atnp01* and *atnp02* not treated and treated with 80 mg L^-1^ CdS QDs.


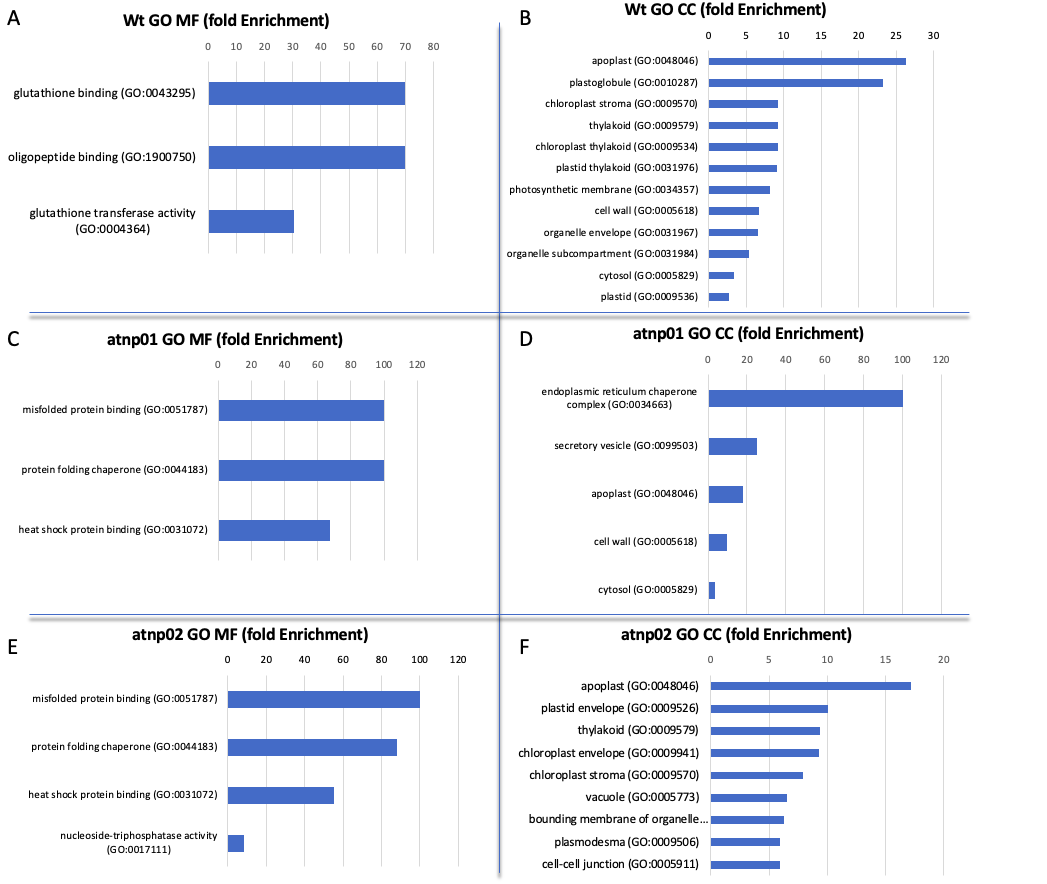


Figure S6. Gene Ontology and enrichment analyses with fold enrichment = -log10 (Fisher’s exact p value) for **(a)** molecular function wt; **(b)** cellular component wt; **(c)** molecular function *atnp01,* **(d)** cellular component *atnp01*; **(e)** molecular function *atnp02,* **(f)** cellular component *atnp02.*


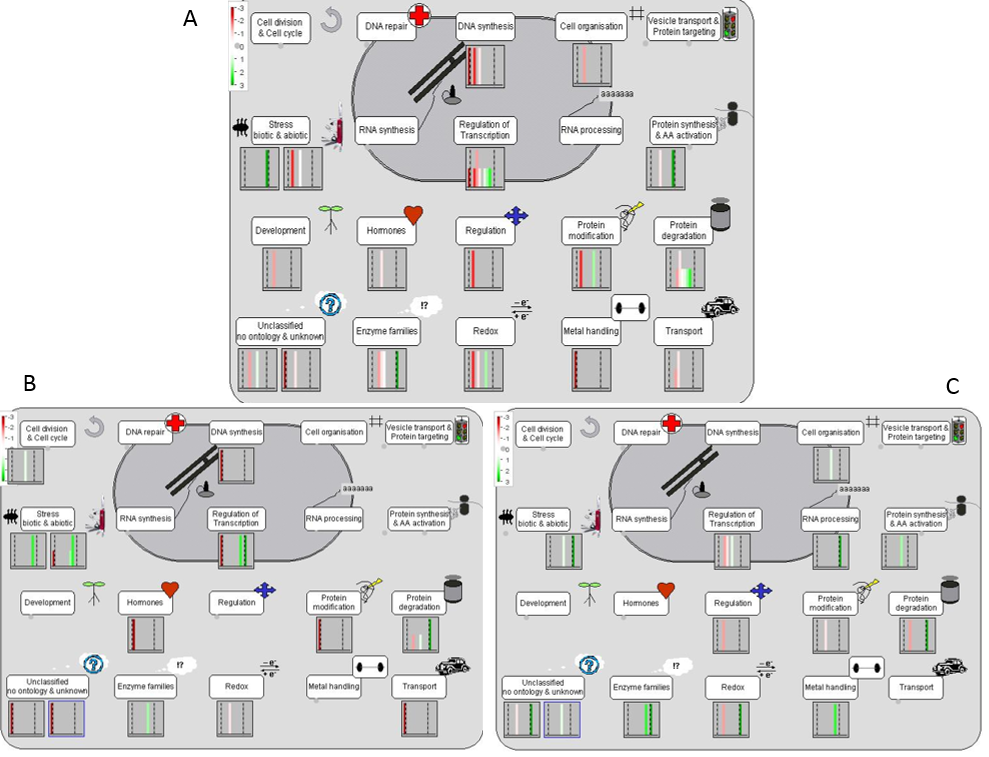


Figure S7. Cell function overview map after CdS QDs exposure. Cell functions associated with the proteomic changes affecting *Arabidopsis thaliana* after CdS QDs exposure in wt **(a)** *atnp01* **(b)** and in *atnp02* **(c)** using MapMan software. The represented squares are only for proteins showing a significant change in protein abundance between the treatment and the untreated control that were attributed to the respective bins by MapMan. Up and down regulated proteins are indicated in red and green, respectively.


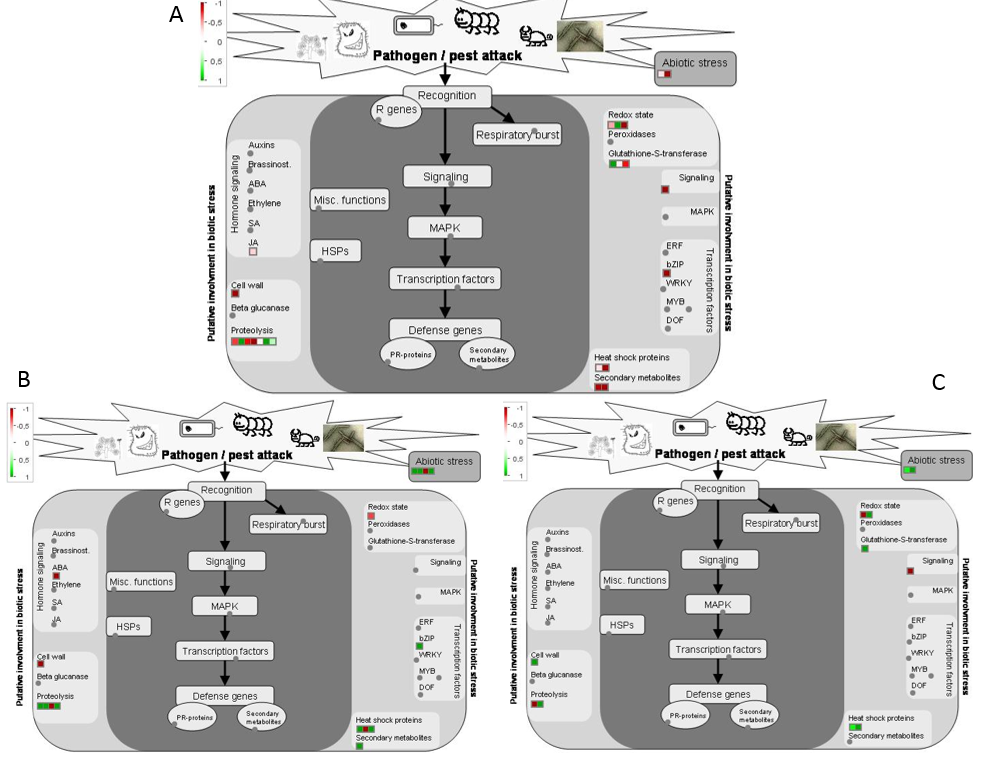


Figure S8. Biotic stress overview map after CdS QDs exposure. Stress response associated with the proteomic changes affecting *A. thaliana* after CdS QDs exposure in wt **(a)** *atnp01* **(b)** and in *atnp02* **(c)** using MapMan software. The represented squares are only for proteins showing a significant change in protein abundance between the treatment and the untreated control that were attributed to the respective bins by MapMan. Up and down regulated proteins are indicated in red and green, respectively.

### Table S2. MapMan BIN assignation and description of differentially abundant proteins in *Arabidopsis thaliana.*

| Protein name | bin code | bin name | description |
| --- | --- | --- | --- |
| 30S ribosomal protein S5, chloroplastic | 29.2.1.1.1.1.5 | protein.synthesis.ribosomal protein.prokaryotic.chloroplast.30S subunit.S5 | Binds directly to 16S ribosomal RNA. |
| 3-isopropylmalate dehydratase small subunit 3 | 16.5.1.1.1.4 | secondary metabolism.sulfur-containing.glucosinolates.synthesis.aliphatic.methylthioalkylmalate isomerase small subunit (MAM-IS) | Catalyzes the isomerization between 2-isopropylmalate and 3-isopropylmalate, via the formation of 2-isopropylmaleate. Plays an essential role in leucine biosynthesis and female gametophyte development. |
| Aminomethyltransferase, mitochondrial | 1.2.4.2  13.2.5.2 | PS.photorespiration.glycine cleavage.T subunit  amino acid metabolism.degradation.serine-glycine-cysteine group.glycine | The glycine decarboxylase (GDC) or glycine cleavage system catalyzes the degradation of glycine. |
| Aspartokinase 2, chloroplastic | 13.1.3.6.1.1 | amino acid metabolism.synthesis.aspartate family.misc.homoserine.aspartate kinase | Involved in the first step of essential amino acids lysine, threonine, methionine and isoleucine synthesis via the aspartate-family pathway. |
| ATP synthase subunit beta-1, mitochondrial | 1.2.4.2  13.2.5.2 | PS.photorespiration.glycine cleavage.T subunit  amino acid metabolism.degradation.serine-glycine-cysteine group.glycine | Mitochondrial membrane ATP synthase (F1F0 ATP synthase or Complex V) produces ATP from ADP in the presence of a proton gradient across the membrane which is generated by electron transport complexes of the respiratory chain. Subunits alpha and beta form the catalytic core in F1. |
| ATP-dependent helicase BRM | 27.3.44 | RNA.regulation of transcription.chromatin remodelling factors | ATPase subunit of a multiprotein complex equivalent of the SWI/SNF complex that acts by remodeling the chromatin by catalyzing an ATP-dependent alteration in the structure of nucleosomal DNA. |
| ATP-dependent zinc metalloprotease FTSH 2 | 29.5.7 | protein.degradation.metalloprotease | Part of a complex that function as an ATP-dependent zinc metallopeptidase. Involved in the thylakoid formation and in the removal of damaged D1 in the photosystem II, preventing cell death under high-intensity light conditions, but not involved in thermotolerance. |
| Auxin-responsive protein IAA9 | 17.2.3 | hormone metabolism.auxin.induced-regulated-responsive-activated | Aux/IAA proteins are short-lived transcriptional factors that function as repressors of early auxin response genes at low auxin concentrations. |
| Bifunctional enolase 2/transcriptional activator | 4.1.13 | glycolysis.cytosolic branch.enolase | Multifunctional enzyme that acts as an enolase involved in the metabolism and as a positive regulator of cold-responsive gene transcription. |
| Catalase-2 | 21.6 | redox.dismutases and catalases | Occurs in almost all aerobically respiring organisms and serves to protect cells from the toxic effects of hydrogen peroxide. |
| Chaperone protein ClpC2, chloroplastic | 29.5.5 | protein.degradation.serine protease | Molecular chaperone. Has an ATPase activity, but no ADPase activity. |
| Chaperonin 60 subunit alpha 1, chloroplastic | 1.3.13  29.6 | PS.calvin cycle.rubisco interacting  protein.folding | Binds RuBisCO small and large subunits and is implicated in the assembly of the enzyme oligomer. Involved in protein assisted folding. Required for proper chloroplast development. |
| Chaperonin 60 subunit beta 1, chloroplastic | 1.3.13  29.6 | PS.calvin cycle.rubisco interacting  protein.folding | Binds RuBisCO small and large subunits and is implicated in the assembly of the enzyme oligomer. Involved in protein assisted folding. Required for proper plastid division. |
| Chloroplast stem-loop binding protein of 41 kDa a | 27.3.99  31.4 | RNA.regulation of transcription.unclassified  cell.vesicle transport | Binds and cleaves RNA, particularly in stem-loops. Associates with pre-ribosomal particles in chloroplasts, and participates in chloroplast ribosomal RNA metabolism. Required for chloroplast integrity. Involved in the regulation of the circadian system. |
| Cullin-3B | 29.5.11.4.3.3  29.5.11.4.5.1 | protein.degradation.ubiquitin.E3.SCF.cullin  protein.degradation.ubiquitin.E3.BTB/POZ Cullin3.Cullin3 | Component of the cullin-RING ubiquitin ligases (CRL). The functional specificity of the CRL complex depends on the BTB domain-containing protein as the susbstrate recognition component. |
| Elongator complex protein 3 | 27.3.54 | RNA.regulation of transcription.histone acetyltransferases | Histone acetyltransferase component of the large multiprotein complex Elongator that is involved in the regulation of transcription initiation and elongation. |
| Eukaryotic translation initiation factor 5A-2 | 29.2.3 | protein.synthesis.initiation | Bimodular protein capable of binding to both RNA and proteins. Regulates cytokinin-mediated root. Regulates the induction of programmed cell death caused by infection with virulent pathogen. |
| F-box only protein 7 | 29.5.11.4.3.2 | protein.degradation.ubiquitin.E3.SCF.FBOX | Confers specificity to the E3 ligase through direct physical interactions with the degradation substrate |
| F-box/kelch-repeat protein At4g19865 | 29.5.11.4.3.2 | protein.degradation.ubiquitin.E3.SCF.FBOX | Confers specificity to the E3 ligase through direct physical interactions with the degradation substrate |
| F-box/LRR-repeat protein At2g40920 | 29.5.11.4.3.2 | protein.degradation.ubiquitin.E3.SCF.FBOX | Confers specificity to the E3 ligase through direct physical interactions with the degradation substrate |
| Filament-like plant protein 6 |  |  | Member of the filament-like plant protein (FPP) family |
| Folylpolyglutamate synthase | 19.4    25.8 | tetrapyrrole synthesis.ALA dehydratase  C1-metabolism.tetrahydrofolate synthase | Catalyzes conversion of folates to polyglutamate. Essential for organellar and whole-plant folate homeostasis. Required for postembryonic root development. Generates polyglutamylated folate cofactors to support C1 metabolism required for meristem maintenance and cell expansion during postembryonic root development |
| Formate--tetrahydrofolate ligase | 25.2  27.3.44 | C1-metabolism.formate-tetrahydrofolate ligase  RNA.regulation of transcription.chromatin remodeling factors | This protein is involved in the pathway tetrahydrofolate interconversion, which is part of One-carbon metabolism.View all proteins of this organism that are known to be involved in the pathway [tetrahydrofolate interconversion](http://www.uniprot.org/uniprot/?query=organism:3702+pathway:445.193) and in One-carbon metabolism |
| Fumarate hydratase 1 | 8.1.8 | TCA / organic transformation.TCA.fumarase | This protein is involved in step of the subpathway that synthesizes (S)-malate from fumarate. |
| Gamma carbonic anhydrase 2 | 9.1.1.5  21.2.2 | mitochondrial electron transport / ATP synthesis.NADH-DH (type I).complex I.carbonic anhydrase  redox.ascorbate and glutathione.glutathione | Enzyme involved in the catabolism of H_2_CO_3_ but that does not mediates the reversible hydration of carbon dioxide. Mediates complex I assembly in mitochondria and respiration. |
| Glutamate--glyoxylate aminotransferase 1 | 13.1.1.3.1    13.1.5.2.2 | amino acid metabolism.synthesis.central amino acid metabolism.alanine.alanine aminotransferase  amino acid metabolism.synthesis.serine-glycine-cysteine group.glycine.glycine transaminase | Catalyzes the glutamate:glyoxylate (GGT or GGAT), alanine:glyoxylate (AGT), alanine:2-oxoglutarate (AKT) and glutamate:pyruvate (GPT) aminotransferase reactions in peroxisomes. Required for abscisic acid (ABA)- and stress-mediated responses in an H_2_O_2_-dependent manner. Function as a photorespiratory aminotransferase that modulates amino acid content during photorespiration (GGAT activity); promotes serine, glycine and citrulline metabolism in response to light |
| Glutamine synthetase, chloroplastic/mitochondrial | 12.2.2 | N-metabolism.ammonia metabolism.glutamine synthetase | The light-modulated chloroplast/mitochondrial enzyme, encoded by a nuclear gene and expressed primarily in leaves, is responsible for the reassimilation of the ammonia generated by photorespiration. |
| Glutathione gamma-glutamylcysteinyltransferase 2 | 15.2 | metal handling.binding, chelation and storage | Involved in the synthesis of phytochelatins (PC) and homophytochelatins (hPC), the heavy-metal-binding peptides of plants. |
| Glutathione S-transferase DHAR3, chloroplastic | 21.2.1 | redox.ascorbate and glutathione.ascorbate | Exhibits glutathione-dependent thiol transferase and dehydroascorbate (DHA) reductase activities. Key component of the ascorbate recycling system. Involved in the redox homeostasis, especially in scavenging of ROS under oxidative stresses. |
| Glutathione S-transferase F2 | 26.9 | misc.glutathione S transferases | Binds auxin, endogenous flavonoids and the phytoalexin camalexin and may be involved in regulating the binding and transport of small bioactive natural products and defense-related compounds during plant stress. Acts as glutathione peroxidase on cumene hydroperoxide, linoleic acid-13-hydroperoxide and trans-stilbene oxide. |
| Glutathione S-transferase F3 | 26.9 | misc.glutathione S transferases | Binds a series of heterocyclic compounds, including lumichrome, harmane, norharmane and indole-3-aldehyde. May be involved in the conjugation of reduced glutathione to a wide number of exogenous and endogenous hydrophobic electrophiles and have a detoxification role against certain herbicides. |
| Glutathione S-transferase F7 | 26.9 | misc.glutathione S transferases | May be involved in the conjugation of reduced glutathione to a wide number of exogenous and endogenous hydrophobic electrophiles and have a detoxification role against certain herbicides. |
| Glutathione S-transferase F9 | 26.9 | misc.glutathione S transferases | May be involved in the conjugation of reduced glutathione to a wide number of exogenous and endogenous hydrophobic electrophiles and have a detoxification role against certain herbicides. |
| Glyceraldehyde-3-phosphate dehydrogenase GAPA1, chloroplastic | 1.3.4 | PS.calvin cycle.GAP | Involved in the photosynthetic reductive pentose phosphate pathway (Calvin-Benson cycle). Catalyzes the reduction of 1,3-diphosphoglycerate by NADPH |
| Glyceraldehyde-3-phosphate dehydrogenase GAPA2, chloroplastic | 1.3.4 | PS.calvin cycle.GAP | Involved in the photosynthetic reductive pentose phosphate pathway (Calvin-Benson cycle). Catalyzes the reduction of 1,3-diphosphoglycerate by NADPH |
| Glyceraldehyde-3-phosphate dehydrogenase GAPB, chloroplastic | 1.3.4 | PS.calvin cycle.GAP | Involved in the photosynthetic reductive pentose phosphate pathway (Calvin-Benson cycle). Catalyzes the reduction of 1,3-diphosphoglycerate by NADPH |
| Glyceraldehyde-3-phosphate dehydrogenase GAPC1, cytosolic | 4.1.8 | glycolysis.cytosolic branch.glyceraldehyde 3-phosphate dehydrogenase (GAP-DH) | Key enzyme in glycolysis that catalyzes the first step of the pathway by converting D-glyceraldehyde 3-phosphate (G3P) into 3-phospho-D-glyceroyl phosphate. Essential for the maintenance of cellular ATP levels and carbohydrate metabolism. Involved in response to oxidative stress by mediating plant responses to abscisic acid (ABA) and water deficits May be part of a redox-dependent retrograde signal transduction network for adaptation upon oxidative stress. |
| Glycine dehydrogenase (decarboxylating) 2 | 1.2.4.1 | PS.photorespiration.glycine cleavage.P subunit | The glycine decarboxylase (GDC) catalyzes the degradation of glycine. |
| Glycine-rich RNA-binding protein 7 | 27.4 | RNA binding | Plays a role in RNA transcription or processing during stress. Binds RNAs and DNAs sequence with a preference to single-stranded nucleic acids. |
| Glycine-rich RNA-binding protein 8 | 27.4 | RNA.RNA binding | Plays a role in RNA transcription or processing during stress. Binds RNAs and DNAs sequence with a preference to single-stranded nucleic acids. Involved in mRNA alternative splicing of numerous targets by modulating splice site selection. |
| Heat shock 70 kDa protein 10, mitochondrial | 20.2.1 | stress.abiotic.heat | In cooperation with other chaperones, Hsp70s stabilize preexistent proteins against aggregation and mediate the folding of newly translated polypeptides in the cytosol as well as within organelles. |
| Heat shock 70 kDa protein 3 | 20.2.1  29.6 | stress.abiotic.heat  protein.folding | In cooperation with other chaperones, Hsp70s stabilize preexistent proteins against aggregation and mediate the folding of newly translated polypeptides in the cytosol as well as within organelles. |
| Histone H1.1 | 28.1.3 | DNA.synthesis/chromatin structure.histone | Histones H1 are necessary for the condensation of nucleosome chains into higher-order structures. |
| Histone H2B.7 | 28.1.3 | DNA.synthesis/chromatin structure.histone | Core component of nucleosome. Nucleosomes wrap and compact DNA into chromatin Histones there by play a central role in transcription regulation, DNA repair, DNA replication and chromosomal stability. |
| Jacalin-related lectin 33 | 26.16 | misc.myrosinases-lectin-jacalin | Sugar-binding protein showing significant affinity for maltohexaose, isomaltohexaose. |
| Kinesin-5 | 31.1 | cell.organisation | ATP binding and microtubule binding |
| L-ascorbate peroxidase 1, cytosolic | 21.2.1 | redox.ascorbate and glutathione.ascorbate | Plays a key role in hydrogen peroxide removal. Constitutes a central component of the reactive oxygen gene network. |
| Magnesium-chelatase subunit ChlI-2 | 19.10 | tetrapyrrole synthesis.magnesium chelatase | Involved in chlorophyll biosynthesis. Catalyzes the insertion of magnesium ion into protoporphyrin IX to yield Mg-protoporphyrin IX. |
| Mediator of RNA polymerase II transcription subunit 37a | 20.2.1  29.6 | stress.abiotic.heat  protein.folding | Component of the Mediator complex, a coactivator involved in the regulated transcription of nearly all RNA polymerase II-dependent genes. |
| Mediator of RNA polymerase II transcription subunit 37F | 20.2.1  29.6 | stress.abiotic.heat  protein.folding | Component of the Mediator complex, a coactivator involved in the regulated transcription of nearly all RNA polymerase II-dependent genes. |
| Membrane-associated progesterone-binding protein 4 | 21.2 | redox.ascorbate and glutathione | [heme binding](https://www.ebi.ac.uk/QuickGO/term/GO:0020037) and steroid binding |
| Methyl-CpG-binding domain-containing protein 13 |  |  | Probable transcriptional regulator. |
| Molybdenum cofactor sulfurase | 17.1.1 | hormone metabolism.abscisic acid.synthesis-degradation | Sulfurates the molybdenum cofactor. Sulfation of molybdenum is essential for xanthine dehydrogenase (XDH) and aldehyde oxidase (ADO) enzymes in which molybdenum cofactor is liganded by 1 oxygen and 1 sulfur atom in active form. Modulates cold stress- and osmotic stress-responsive gene expression by acting as key regulator of abscisic acid (ABA) biosynthesis. |
| Myosin-16 | 31.1 | cell.organisation | Myosin heavy chain that is required for the cell cycle-regulated transport of various organelles and proteins for their segregation. Functions by binding with its tail domain to receptor proteins on organelles and exerting force with its N-terminal motor domain against actin filaments. |
| Myrosinase 1 | 16.5.1.3.1.1 | secondary metabolism.sulfur-containing.glucosinolates.degradation.myrosinase.TGG | Degradation of glucosinolates to glucose, sulfate and any of the products: thiocyanates, isothiocyanates, nitriles, epithionitriles or oxazolidine-2-thiones. Seems to function in abscisic acid (ABA) and methyl jasmonate (MeJA) signaling in guard cells. |
| NifU-like protein 3 | 29.8 | protein.assemby and cofactor ligation | Molecular scaffold for [Fe-S] cluster assembly of chloroplastic iron-sulfur proteins. |
| Oxygen-evolving enhancer protein 1-1, chloroplastic | 1.1.1.2 | PS.lightreaction.photosystemII.PSII polypeptide subunits | Stabilizes the manganese cluster which is the primary site of water splitting. |
| Osmotin-like protein OSM34 | 20.1.7.5 | stress.biotic.PR-proteins.PR5 | defense response to bacterium and fungus.  response to salt stress |
| Patellin-1 | 1.1.1.2 | PS.lightreaction.photosystem II.PSII polypeptide subunits | Carrier protein that may be involved in membrane-trafficking events associated with cell plate formation during cytokinesis. |
| Pathogenesis-related protein 5 | 20.1.7.5 | stress.biotic.PR-proteins.PR5 | Partially responsible for acquired pathogen resistance. |
| Pentatricopeptide repeat-containing protein At1g09220, mitochondrial |  |  | Endonuclease activity |
| Peroxiredoxin-2E, chloroplastic | 21.5 | redox.peroxiredoxin | Plays a role in cell protection against oxidative stress by detoxifying peroxides. May be involved in chloroplast redox homeostasis. |
| Phosphatidylinositol/phosphatidylcholine transfer protein SFH3 | 1.3.12 | PS.calvin cycle.PRK | Required for transport of secretory proteins from the Golgi complex. |
| Phosphoribulokinase, chloroplastic | 1.3.4 | PS.calvin cycle.GAP | Protein involved in the pathway Calvin cycle, which is part of Carbohydrate biosynthesis. |
| Plant intracellular Ras-group-related LRR protein 2 |  |  | Leucine-rich repeat protein that likely mediates protein interactions, possibly in the context of signal transduction. |
| Plant UBX domain-containing protein 10 | 29.5 | protein degradation | Biological process, Neutrophil degranulation. |
| Probable fatty acyl-CoA reductase 4 | 11.9.4.13 | lipid metabolism.lipid degradation.beta-oxidation.acyl CoA reductase | Catalyzes the reduction of fatty acyl-CoA to fatty alcohols. Provides the fatty alcohols required for synthesis of suberin in roots, seed coat and wound-induced leaf tissue. Provides the fatty alcohols required for synthesis of alkyl hydroxycinnamates in root waxes. |
| Probable fructose-bisphosphate aldolase 2 | 1.3.6 | PS.calvin cycle.aldolase | Plays a key role in glycolysis and gluconeogenesis. |
| Probable inactive ATP-dependent zinc metalloprotease FTSHI 1 | 29.5.7 | protein.degradation.metalloprotease | Functions in chloroplast biogenesis and chloroplast division |
| Probable mediator of RNA polymerase II transcription sub 37c | 20.2.1  29.6 | stress.abiotic.heat  protein.folding | Component of the Mediator complex, a coactivator involved in the regulated transcription of nearly all RNA polymerase II-dependent genes. |
| Probable mediator of RNA polymerase II transcription sub 37e | 20.2.1  29.6 | stress.abiotic.heat  protein.folding | Component of the Mediator complex, a coactivator involved in the regulated transcription of nearly all RNA polymerase II-dependent genes. In cooperation with other chaperones, Hsp70s stabilize preexistent proteins against aggregation and mediate the folding of newly translated polypeptides in the cytosol as well as within organelles. |
| Probable pectinesterase/pectinesterase inhibitor VGDH2 | 10.8.1 | cell wall.pectinesterases.PME | Acts in the modification of cell walls via demethylesterification of cell wall pectin. |
| Probable protein phosphatase 2C 58 | 29.4 | protein.postranslational modification | Serine/threonine phosphate |
| Probable receptor-like protein kinase At2g47060 | 29.4.1.58 | protein.postranslational modification.kinase.receptor like cytoplasmatic kinase VIII | ATP binding and transmembrane receptor protein serine/threonine kinase activity |
| Prohibitin-3, mitochondrial | 31.2 | cell.division | Prohibitin probably acts as a holdase/unfoldase for the stabilization of newly synthesized mitochondrial proteins. Necessary for mitochondrial and cell metabolism and biogenesis. |
| Protein RETICULATA | 33.99 | development.unspecified | May play a role in leaf development. |
| Protein TIFY 8 |  |  | Repressor of jasmonate responses. |
| Putative F-box protein At2g19630 | 29.5.11.4.3.2 | protein.degradation.ubiquitin.E3.SCF.FBOX | Confers specificity to the E3 ligase through direct physical interactions with the degradation substrate |
| Putative F-box/LRR-repeat protein At4g13960 | 29.5.11.4.3.2 | protein.degradation.ubiquitin.E3.SCF.FBOX | Confers specificity to the E3 ligase through direct physical interactions with the degradation substrate |
| Putative RING-H2 finger protein ATL49 | 29.5.11.4.3.2 | protein.degradation.ubiquitin.E3.SCF.FBOX | May be involved in female gametophyte development. |
| PYK10-binding protein 1 | 26.16 | misc.myrosinases-lectin-jacalin | Inhibitor-type lectin that may regulate the correct polymerization of BGLU23/PYK10 upon tissue damage. |
| Pyrophosphate fructose6-phosph1-phosphotransferase sub beta1 | 4.2.5 | glycolysis.plastid branch.pyrophosphate-fructose-6-P phosphotransferase | Catalytic subunit of pyrophosphate--fructose 6-phosphate 1-phosphotransferase. Catalyzes the phosphorylation of D-fructose 6-phosphate, the first committing step of glycolysis. Uses inorganic phosphate (PPi) as phosphoryl donor instead of ATP like common ATP-dependent phosphofructokinases (ATP-PFKs), which renders the reaction reversible, and can thus function both in glycolysis and gluconeogenesis. |
| Ras-related protein RABF1 | 30.5 | signalling.G-proteins | Endosomal protein probably involved in endocytosis. Probably not involved in vacuolar trafficking. |
| Ribulose bisphosphate carboxylase large chain | 1.3.1  29.2.1.1.1.15 | PS.calvin cycle.rubisco large subunit protein.synthesis.ribosomal protein.prokaryotic.chloroplast.30S subunit.S15 | RuBisCO catalyzes two reactions: the carboxylation of D-ribulose 1,5-bisphosphate, the primary event in carbon dioxide fixation, as well as the oxidative fragmentation of the pentose substrate in the photorespiration process. |
| Ribulose bisphosphate carboxylase/oxygenase activase | 1.3.13 | PS.calvin cycle.rubisco interacting | Activation of RuBisCO (ribulose-1,5-bisphosphate carboxylase/oxygenase; involves the ATP-dependent carboxylation of the epsilon-amino group of lysine leading to a carbamate structure. |
| S-adenosylmethionine synthase 1 | 13.1.3.4.11 | amino acid metabolism.synthesis.aspartate family.methionine.S-adenosylmethionine synthetase | Catalyzes the formation of S-adenosylmethionine from methionine and ATP. |
| Serine/arginine-rich SC35-like splicing factor SCL28 | 27.1.1 | RNA.processing.splicing | Involved in intron recognition and spliceosome assembly |
| Sorbitol dehydrogenase |  |  | Converts sorbitol to fructose. Mostly active with sorbitol, ribitol and xylitol as substrates. |
| Terpenoid synthase 25 | 16.1.5 | secondary metabolism.isoprenoids.terpenoids | This protein is involved in the pathway terpenoid biosynthesis, which is part of Secondary metabolite biosynthesis. |
| Thylakoid lumenal protein TL20.3 | 29.5 | protein.degradation | Pentapeptide repeat protein of unknown function. Subject to degradation when reduced. |
| Transcription factor TGA4 | 27.3.35 | RNA.regulation of transcription.bZIP transcription factor family | Transcriptional activator that binds specifically to the DNA sequence 5'-TGACG-3'. Recognizes ocs elements like the as-1 motif of the cauliflower mosaic virus 35S promoter. Binding to the as-1-like cis elements mediate auxin- and salicylic acid-inducible transcription. May be involved in the induction of the systemic acquired resistance (SAR) via its interaction with NPR1. Could also bind to the Hex-motif (5'-TGACGTGG-3') another cis-acting element found in plant histone promoters. |
| Triosephosphate isomerase, cytosolic | 1.3.5  4.1.7 | PS.calvin cycle.TPI  glycolysis.cytosolic branch.triosephosphate isomerase (TPI) | This protein is involved in the pathway gluconeogenesis, which is part of Carbohydrate biosynthesis. |
| tRNA-splicing endonuclease subunit Sen2-1 | 27.1.1 | RNA.processing.splicing | Constitutes one of the two catalytic subunits of the tRNA-splicing endonuclease complex, a complex responsible for identification and cleavage of the splice sites in pre-tRNA. |
| UDP-glucuronate 4-epimerase 5 | 10.1.6 | cell wall.precursor synthesis.GAE | Involved in the synthesis of the negatively charged monosaccharide that forms the backbone of pectic cell wall components. |
| Vacuolar protein sorting-associated protein 24 homolog 1 | 27.3.71 | RNA.regulation of transcription.SNF7 | Component of the ESCRT-III complex, which is required for multivesicular bodies (MVBs) formation and sorting of endosomal cargo proteins into MVBs. |
| V-type proton ATPase subunit B1 | 34.1.1.1 | transport.p- and v-ATPases.H+-transporting two-sector ATPase.subunit B | Non-catalytic subunit of the peripheral V1 complex of vacuolar ATPase. V-ATPase is responsible for acidifying a variety of intracellular compartments in eukaryotic cells. |
| V-type proton ATPase subunit B2 | 34.1.1.1 | transport.p- and v-ATPases.H+-transporting two-sector ATPase.subunit B | Non-catalytic subunit of the peripheral V1 complex of vacuolar ATPase. V-ATPase is responsible for acidifying a variety of intracellular compartments in eukaryotic cells. |
| Zinc finger A20 and AN1 domain-containing stress-assoc prot 1 | 27.3.11 | RNA.regulation of transcription. C2H2 zinc finger family | May be involved in environmental stress response. |
